# Plasma and Brain Metabolomics Uncover Modulation of Bile Acid and Pentose Phosphate Pathways by *Melissa officinalis* in Obese Rat Model

**DOI:** 10.3390/ijms27052391

**Published:** 2026-03-04

**Authors:** Fatima Zohra Aberkane, Laura Natalia Ferro Holguín, Anne-Sophie Roy, Claire Maltret, Sekhou Cisse, Mohammed El Amine Benarbia, Séverine Boisard, Mohamed Yassine Mallem, David Guilet

**Affiliations:** 1 Univ Angers, SONAS, SFR QUASAV, 49000 Angers, France; lauranatalia.ferroholguin@univ-angers.fr (L.N.F.H.); severine.boisard@univ-angers.fr (S.B.); 2 Labcom FeedInTech, 42 Rue Georges Morel, 49070 Beaucouzé, France; claire.maltret@norfeed.net (C.M.); sekhou.cisse@norfeed.net (S.C.); amine.benarbia@norfeed.net (M.E.A.B.); 3 Nor-Feed SAS, 3 Rue Amedeo Avogadro, 49070 Beaucouzé, France; 4 Nutrition, PathoPhysiology and Pharmacology (NP3) Unit, 101 Rte de Gachet, Oniris, 44300 Nantes, France; anne.sophie.roy.93@gmail.com (A.-S.R.); yassine.mallem@oniris-nantes.fr (M.Y.M.)

**Keywords:** stress, behavior, metabolomics

## Abstract

While our group previously demonstrated the calming effects of *Melissa officinalis* extract (MOE) in dogs, the underlying brain-level mechanisms remain unclear. To address this, we investigated these mechanisms in rats using an untargeted metabolomics approach. Twenty-four male Wistar rats were divided into three groups (eight rats per group): control (standard diet, SD), a group fed a high-fat high-sucrose diet (HFHSD), and HFHSD administrated with a hydro-alcoholic standardized MOE (HFHSD MOE) at a dose of 200 mg/kg. Body weight, behavior through elevated plus maze (EPM), and glucose tolerance using the oral glucose tolerance test (OGTT) were monitored. After 12 weeks of supplementation, plasma and brain metabolomes were explored using non-targeted metabolomics. Although the EPM revealed no significant behavioral improvement, the OGTT showed a significant reduction in blood glucose area under the curve (AUC, *p* < 0.05), suggesting a metabolic effect of MOE. Metabolomic analysis highlighted two key pathways: (1) bile acid biosynthesis in plasma, as previously observed in our dog study, and (2) pentose phosphate metabolism in the brain. These results provide insight into central and peripheral mechanisms influenced by MOE and generate hypotheses on pathways potentially linked to previously reported behavioral effects in dogs, offering targets for nutritional interventions.

## 1. Introduction

In dogs, chronic stress is a leading contributor to behavioral disorders [1]. A prolonged exposition to stressors such as loud noise and spatial and social restriction alters dogs’ well-being and quality of life [1,2]. In ethnoveterinary practice, plant-based additives are among the approaches used to help manage these disorders in dogs [3,4]. Of these, *Melissa officinalis* extract (MOE) is well-documented for its calming and anxiolytic effects in both animals and humans [5,6]. Although the anxiolytic effects of MOE have been widely demonstrated, the underlying mechanisms are not always highlighted.

In cases where mechanisms are investigated, hypothesis-driven research is often employed to test and validate hypotheses regarding these mechanisms. For instance, one study investigated the anxiolytic effects of a *Melissa officinalis* extract by examining its ability to modulate acetylcholine receptors in the human occipital cortex tissue [7]. Another study focused on the inhibitory neurotransmitter γ-aminobutyric acid (GABA), demonstrating that rosmarinic acid inhibits the enzyme GABA transaminase (GABA-T), thereby increasing GABA levels and leading to a decrease in anxiety [8]. In the same vein, Ghazizadeh et al. reported that the anxiolytic effect of MOE is due to its antioxidant and anti-apoptotic capacities [9].

While hypothesis-driven research is a powerful and robust approach, focusing on specific, targeted aspects, broad-spectrum exploratory approaches permit a global overview of complex systems, enabling the identification of unexpected biomarkers and providing new insights into mechanisms of action. In this context and building on our own previous investigation of MOE in dogs, the present study was designed as a follow-up. In that earlier work, conducted by our team, MOE had demonstrated an anxiolytic effect, assessed using a standardized evaluation grid developed at ONIRIS. Additionally, analysis of dogs’ plasma metabolome highlighted several impacted pathways primarily related to bile acids biosynthesis, revealing interesting, unexpected potential biomarkers that may be associated with MOE’s mechanism of action underlying its anxiolytic effects [10].

Due to the impossibility of performing brain sampling in dogs, researchers commonly rely on validated rodent models to examine mechanistic hypotheses emerging from canine studies [11,12]. For this purpose, rats were chosen as an experimental model in this study to allow access to brain tissue for analysis. To increase the sensitivity of this model, and because metabolic disorders are known to enhance susceptibility to stress-related behaviors, a subset of rats was subjected to a diet-induced obesity protocol. Obesity is indeed associated with increased vulnerability to anxiety and depression-like behaviors in rodents [13,14,15].

In this context, this study aimed to evaluate the effects of a hydro-alcoholic MOE supplementation on obese rats, focusing on the hypothalamus to provide deeper insights into the mode of action of MOE in relation to its anxiolytic effect. The effects of MOE were first assessed on body weight, behavior, and glucose tolerance in rats fed a high-fat diet. Subsequently, exploratory research employing a non-targeted metabolomic approach on plasma and hypothalamus tissues was conducted to identify potential biomarkers and highlight the metabolic pathways affected by the supplementation.

## 2. Results

### 2.1. Obesity Model Validation

**Figure 1:**
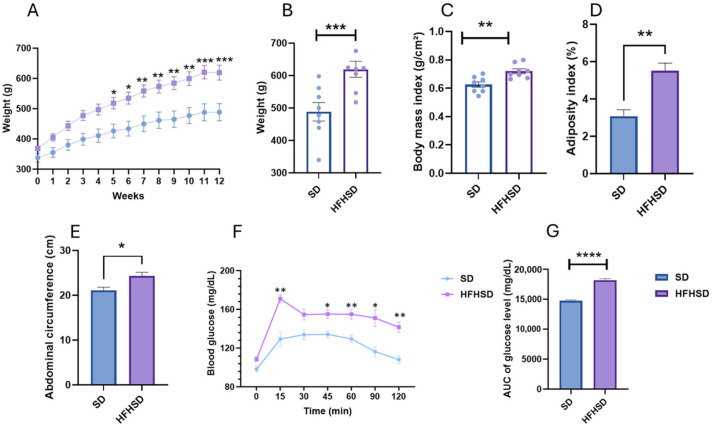
Analysis of diet-induced obesity parameters in rats following 12 weeks of high-fat high-sucrose diet (HFHSD) feeding. (**A**) Body weight monitoring during HFHSD consumption. (**B**) Final body weight and (**C**) body mass index (BMI) values of both groups (SD vs. HFHSD) at the end of the procedure. (**D**) Effects of HFHSD in rats on adiposity index and (**E**) abdominal circumference. (**F**) Oral glucose tolerance test (OGTT) performed on the HFHSD group compared to SD group. (**G**) Area under the curve (AUC) of the HFHSD group compared to SD group. Data are means ± SEMs. * *p* < 0.05; ** *p* < 0.01; *** *p* < 0.001; **** *p* < 0.0001.

In this study, rats were fed an high-fat high-sucrose diet (HFHSD) for 12 weeks to assess the effect of MOE on weight gain, anxiety, and glucose tolerance. First, various parameters were measured to confirm the validity of the obesity model. The results revealed that the HFHSD affected body weight throughout the entire diet period, with a significant increase observed starting from week 5 (*p* < 0.05) and continuing until the end of the diet at week 12 (*p* < 0.005) (Figure 1A). The final body weight of HFHSD rats increased by 29% compared to their initial body weight, significantly exceeding that of the standard diet group (SD) (*p* < 0.005) (Figure 1B). Furthermore, the administration of the HFHSD in rats led to significant increases in the body mass index (BMI) (*p* < 0.01) (Figure 1C), adiposity index (AI) (*p* < 0.01) (Figure 1D), and abdominal circumference (AC) (*p* < 0.05) (Figure 1E) compared to the SD group. Moreover, blood glucose levels in HFHSD rats increased to approximately 170 mg/dL within 15 min of glucose administration (*p* < 0.01). Although these levels later decreased and stabilized around 150 mg/dL at 120 min (*p* < 0.01), they remained elevated compared to the SD group (Figure 1F). The HFHSD rats also demonstrated greater area under the curve compared to the SD group (*p* < 0.0001) (Figure 1G). Collectively, these findings confirm that the 12-week HFHSD protocol successfully established a robust model of obesity with the expected metabolic alterations (i.e., impaired glucose tolerance), thereby providing a solid framework for subsequent investigations into the potential beneficial effects of MOE within this context. 

### 2.2. Impact of MOE on Body Weight

**Figure 2:**
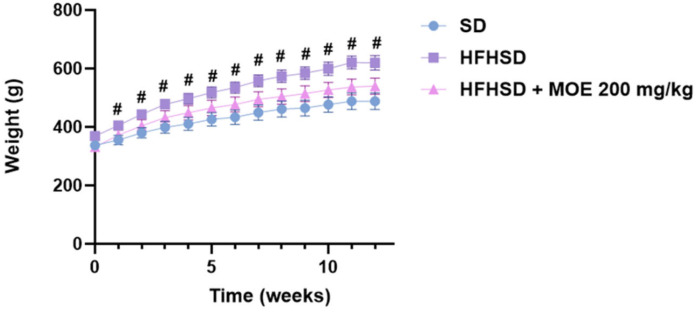
Effect of MOE on body weight. Data are means ± SEMs. # *p* < 0.05 comparison between SD and HFHSD.

Over the 12-week experiment, body weight monitoring showed a significant increase in the weight of the HFHSD group starting from the first week of the diet (*p* < 0.05). The weight gain was consistent and remained significant throughout the 12 weeks (*p* < 0.05) in comparison to the SD diet group. Comparatively, while the body weight of HFHSD rats supplemented with MOE (HFHSD MOE) appeared to be lower than that of HFHSD rats without supplementation, this difference was not statistically significant (Figure 2).

### 2.3. Behavioral Analysis Using Elevated Plus Maze (EPM)

**Figure 3:**
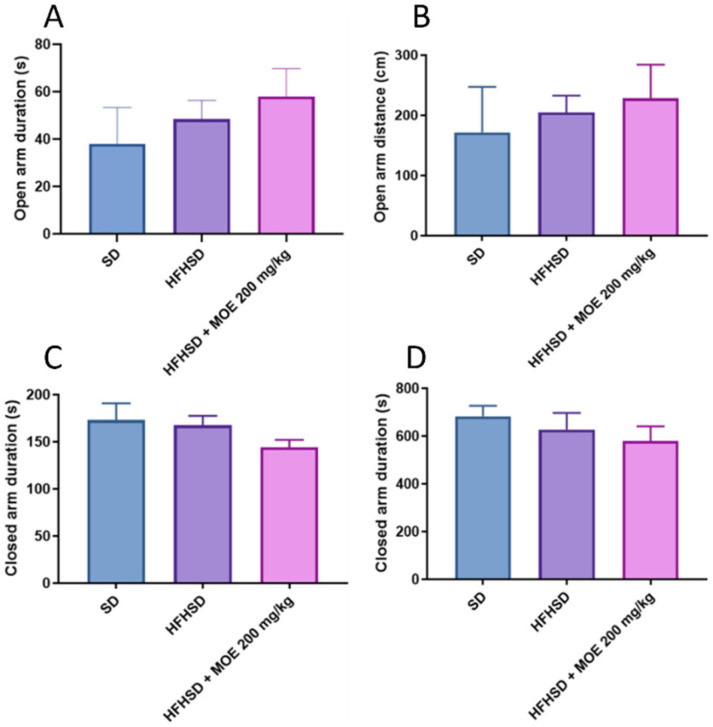
Effects of MOE on anxiety after 12 weeks of supplementation using elevated plus maze (EPM) test. (**A**) Spent duration in the open arms. (**B**) Traveled distance in the open arms. (**C**) Spent duration in the closed arms. (**D**) Traveled distance in the closed arms. Data are means ± SEMs.

Next, the anxiolytic effects of *Melissa officinalis* extract (MOE) were evaluated using HFHSD rats by the Elevated Plus Maze (EPM) after 12 weeks of supplementation. The EPM test used to evaluate anxiety is based on the time spent and the distance traveled in both open and closed arms, with more time in the closed arms indicating higher anxiety, whereas the exploration of the open arms reflects lower anxiety. The results indicated that the HFHSD increased both the duration and distance spent in the open arms while decreasing those in the closed arms; however, these differences were not statistically significant. Likewise, MOE supplementation in HFHSD rats did not produce statistically significant differences in these parameters compared to the HFHSD group (Figure 3).

### 2.4. Effect of MOE on Glucose Homeostasis

**Figure 4:**
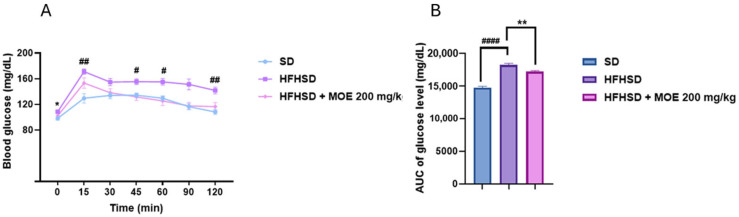
Effect of MOE supplementation on oral glucose tolerance test (OGTT). (**A**) Blood glucose levels (mg/dL) and (**B**) AUC of glucose level (mg/dL) in SD, HFHSD, and HFHSD MOE groups. Data are means ± SEMs. # *p* < 0.05 comparison between SD and HFHSD. ## *p* < 0.01 comparison between SD and HFHSD. #### *p* < 0.0001 comparison between SD and HFHSD. * *p* < 0.05 comparison between HFHSD and HFHSD MOE. ** *p* < 0.01 comparison between HFHSD and HFHSD MOE.

Oral glucose tolerance test (OGTT) results showed that the HFHSD group exhibited elevated blood glucose levels from approximately 15 min onward compared with the standard diet group (*p* < 0.01). This elevation persisted at subsequent time points, remaining significant at 45 (*p* < 0.05), 60 (*p* < 0.05), and 120 min (*p* < 0.01). While supplementation with MOE resulted in a decrease in blood glucose levels at T0 (*p* < 0.05), no significant differences were observed at other time points (Figure 4A). The determination of the AUC showed a significantly higher value in HFHSD rats compared to SD rats (*p* < 0.0001). Supplementation of HFHSD rats with MOE resulted in a reduction in AUC compared to the HFHSD alone (*p* < 0.01) (Figure 4B).

### 2.5. Metabolomics Analysis

#### 2.5.1. Multivariate Analysis

The extraction method demonstrated a broad coverage of metabolites in both hydrophobic extract (HPO) and hydrophilic extract (HPI) extracts that were analyzed in ESI^−^ mode. Then, orthogonal partial least squares–discriminant analysis (OPLS-DA) was performed as a multivariate approach to identify differences between the compared groups. Initially, a comparison between the SD and HFHSD groups was conducted to assess the impact of HFHSD on rats.

**Figure 5:**
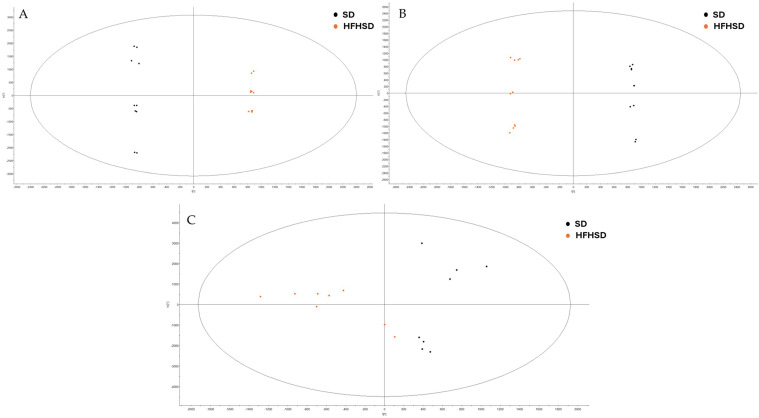
OPLS-DA comparison between standard diet (SD) and high-fat high-sucrose diet (HFHSD) groups. (**A**) Comparison of HPO in ESI^−^ of plasma samples. (**B**) Comparison of HPI in ESI^−^ of plasma samples. (**C**) Comparison of HPO in ESI^−^ of brain samples.

The results demonstrated significant metabolic differences between rats fed an HFHSD and those maintained on a SD. The OPLS-DA of HPO and HPI extracts in brain and plasma samples are shown in Figure 5. Permutation tests were performed to validate these OPLS-DA models. The results indicated that the OPLS-DA models constructed from HPO extracts in both the plasma and brain were not overfitted (Figure S1). Furthermore, OPLS-DA models of HPI extracts in plasma samples were also validated using permutation tests. Nevertheless, for HPI extracts in brain samples, no valid OPLS-DA model could be constructed as the first predictive component was not statistically significant, suggesting that there was insufficient metabolomic-profile-based differentiation between SD and HFHSD groups in case of these extracts. However, for all the other comparisons, permutation tests permitted the validation of the OPLS-DA models (Table S1).

Subsequently, a comparison was conducted between HFHSD rats supplemented with MOE (HFHSD MOE) and those receiving HFHSD alone.

**Figure 6:**
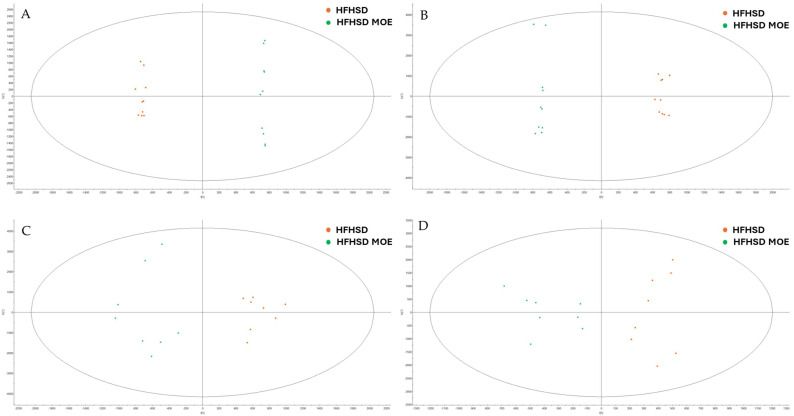
OPLS-DA comparison between control (HFHSD) and supplemented rats (HFHSD MOE). (**A**) Comparison of HPO in ESI^−^ of plasma samples. (**B**) Comparison of HPI in ESI^−^ of plasma samples. (**C**) Comparison of HPO in ESI^−^ of brain samples. (**D**) Comparison of HPI in ESI^−^ of brain samples.

The multivariate approach by OPLS-DA showed a difference between the metabolomes of the HFHSD and HFHSD MOE groups in both HPO and HPI extracts for brain and plasma samples (Figure 6). Permutation tests were also performed to ensure the robustness of the model. The results showed that all the OPLS-DA models were not overfitted and the original test outperformed, mostly, all the permuted tests (Figure S1) (Table S1). These findings reveal that the MOE supplementation of HFHSD rats impacted their metabolome.

#### 2.5.2. The Effects on Metabolic Pathways

- Plasma samples

Two parameters were applied (variable importance in the projection (VIP) > 1 and *p* < 0.05) to identify significantly different features between the compared groups. Following this selection, 1286 features were differently expressed in plasma’s HPO extracts of the HFHSD compared to the SD group. Among these, 113 annotations were accepted based on the identification criteria (Table S2). Concerning HPI extracts, 959 features were differentially expressed. Of them, 136 annotations met the identification criteria (Table S3).

Furthermore, the comparison between HFHSD rats and HFHSD rats supplemented with MOE (HFHSD MOE) revealed 1031 differentially expressed features in plasma, with only 124 meeting the identification criteria in case of HPO extracts (Table S4). Regarding plasma’s HPI extract, 560 features showed a differential expression between the HFHSD and HFSHD MOE. Of these, 84 annotations were accepted (Table S5).

Afterwards, the accepted annotations of plasma samples were consolidated and analyzed using MetaboAnalyst 6.0 through enrichment pathway analysis and pathway analysis based on *Rattus norvegicus*’ from the Kyoto Encyclopedia of Genes and Genomes (KEGG) pathways to gain a deeper understanding of rats’ metabolomes.

**Figure 7:**
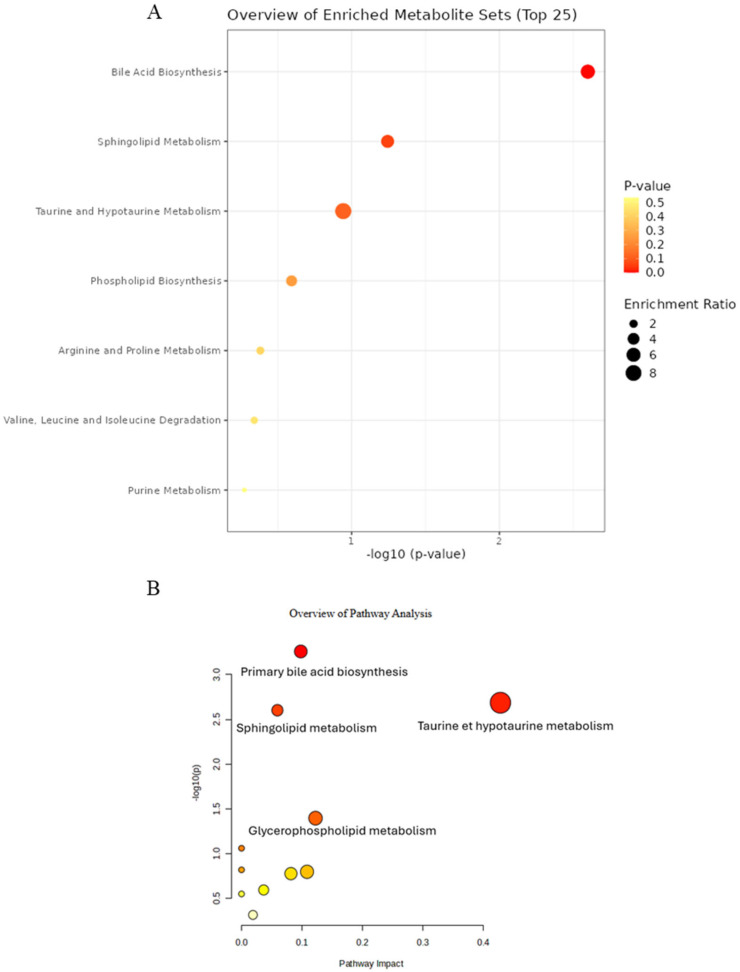
Analysis of impacted metabolic pathways of plasma samples in SD compared to HFHSD group. (**A**) Enrichment pathway using SMPDB integrated into MetaboAnalyst 6.0. (**B**) Pathway analysis using KEGG pathways *of Rattus norvegicus*.

Comparison between SD and HFHSD rats through enrichment pathway analysis using the SMPDB (Small Molecule Pathway DataBase) revelated one impacted pathway in plasma samples: bile acid biosynthesis with four overlapping metabolites (*p* = 2.52 × 10^−3^) (Figure 7A). Other pathways were not considered since the *p*-value was >0.05.

The results from pathway analysis based on *Rattus norvegicus*’ KEGG pathways revealed similar pathways as for the enrichment pathway analysis using the SMPDB. Thoroughly, four pathways were found to be significantly impacted (*p* < 0.05 and impact score > 0): primary bile acid biosynthesis was the most impacted, with four overlapping metabolites (*p* = 5.50 × 10^−4^, impact = 0.098), followed by taurine and hypotaurine metabolism with two hits (*p* = 2.06 × 10^−3^, impact = 0.428), sphingolipid metabolism (*p* = 2.50 × 10^−3^, impact = 0.059) and glycerophospholipid metabolism (*p* = 4.02 × 10^−2^, impact = 0.122), with three and two hits, respectively (Figure 7B). Other pathways were also affected but had either *p* > 0.05 or a pathway impact score of 0 and were therefore excluded.

The overlapped metabolites’ expression profile identified in enrichment and pathway analyses was investigated in plasma samples comparing the SD and HFHSD groups.

Enrichment analysis revealed that three metabolites overlapped on the bile acid biosynthesis pathway (taurocholic acid, taurine, and 3a,7a,12a-Trihydroxy-5b-cholestan-26-al) that were over-expressed in HFHSD rats compared to SD rats. In contrast, glycocholic acid was found to be under-expressed (Table 1).

Similarly, pathway analysis using *Rattus norvegicus*’ KEGG pathways showed the same four metabolites’ profiles for the primary bile acid biosynthesis pathway obtained in enrichment analysis (taurocholic acid, taurine, and 3a,7a,12a-Trihydroxy-5b-cholestan-26-al and glycocholic acid). In taurine/hypotaurine metabolism, both taurine and taurocholic acid were over-expressed in HFHSD rats compared to the SD group. Sphingolipid metabolism exhibited a different metabolite expression: sphingosine 1-phosphate and sphinganine 1-phosphate were over-expressed while ganglioside GM3 (d18:1/9Z-18:1) was under-expressed in HFHSD rats in comparison to SD rats. Then, two overlapped metabolites were found to be impacted in glycerophospholipid metabolism, one under-expressed (LysoPC (18:2/0:0)) and the other over-expressed (PE (14:1(9Z)/20:4(8Z,11Z,14Z,17Z)). These findings, summarized in Table 1, highlight the HFHSD’s impact on rats’ metabolomes (Table 1).

**Figure 8:**
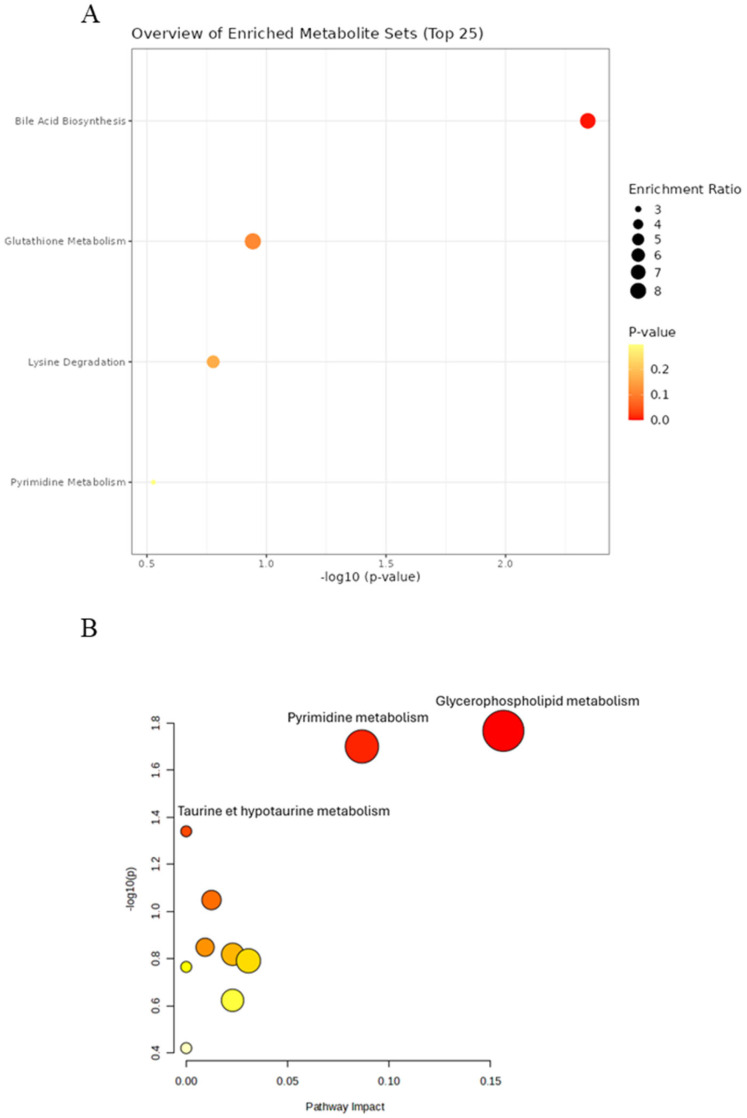
Analysis of impacted metabolic pathways of plasma samples in HFHSD compared to HFHSD MOE group. (**A**) Enrichment pathway using SMPDB integrated into MetaboAnalyst 6.0. (**B**) Pathway analysis using KEGG pathways of *Rattus norvegicus*.

In case where HFHSD rats were supplemented by MOE, the comparison between the HFHSD and HFHSD MOE group revealed different metabolic pathways impacted by MOE supplementation (Figure 8).

In plasma samples, the enrichment analysis showed one significantly impacted pathway: bile acid biosynthesis, with three overlapping metabolites (*p* = 4.53 × 10^−3^) (Figure 8A). On the other hand, using pathway analysis based on *Rattus norvegicus’* KEGG pathways, two pathways were found to be significantly impacted: glycerophospholipid metabolism was the most impacted with two overlapping metabolites (*p* = 1.70 × 10^−2^, impact = 0.156), followed by pyrimidine metabolism, also with two overlapping metabolites, (*p* = 2.00 × 10^−2^, impact = 0.087) (Figure 8B). Other pathways were also affected but had either *p* > 0.05 or a pathway impact score of 0 and were therefore excluded.

Similarly, the expression profiles of overlapping metabolites identified in enrichment and pathway analyses through MetaboAnalyst 6.0 were investigated in plasma samples comparing the HFHSD MOE group to the HFHSD alone (Table 2).

As shown in Table 2, the enrichment analysis of plasma samples highlighted three overlapped metabolites on the bile acid biosynthesis pathway (taurocholic acid, lithocholyltaurine, and taurodeoxycholic acid). These metabolites were under-expressed in the HFHSD MOE group in comparison to the HFHSD alone (Table 2).

Additionally, pathway analysis showed an over-expression of metabolites associated with glycerophospholipid metabolism (LysoPE(0:0/18:1(11Z)) and LysoPA(18:1(9Z)/0:0)) following MOE supplementation. In contrast, the pyrimidine metabolism pathway exhibited the under-expression of dTDP, thymidine 3′,5′-cyclic monophosphate in the HFHSD MOE compared to the HFHSD group alone, as presented in Table 3.

- Brain samples

Following the same methodology, two parameters were applied (VIP > 1 and *p* < 0.05) to identify significantly different features between the compared groups in the case of brain samples. Thoughtfully, the comparison between the HFHSD and SD groups of HPO extracts revealed 93 differently expressed features, but only 25 annotations were accepted (Table S6). Regarding HPI extracts, the permutation tests indicated a lack of metabolomic profile differentiation, leading to their exclusion. Consequently, since the number of differently expressed metabolites between the SD and HFHSD groups was too low, the analysis of metabolic pathways was not feasible. Contrariwise, 165 features were significantly impacted in HPO extracts after the MOE supplementation of HFHSD rats in comparison to HFHSD rats alone, but only 24 annotations were retained (Table S7). Finally, HPI extract analysis revealed 248 differently expressed features. Of them, 28 annotations were accepted based on the identification criteria (Table S8). Therefore, the enrichment analysis of HFHSD rats in comparison to the HFHSHD MOE group of brain samples showed several pathways significantly impacted by MOE supplementation.

**Figure 9:**
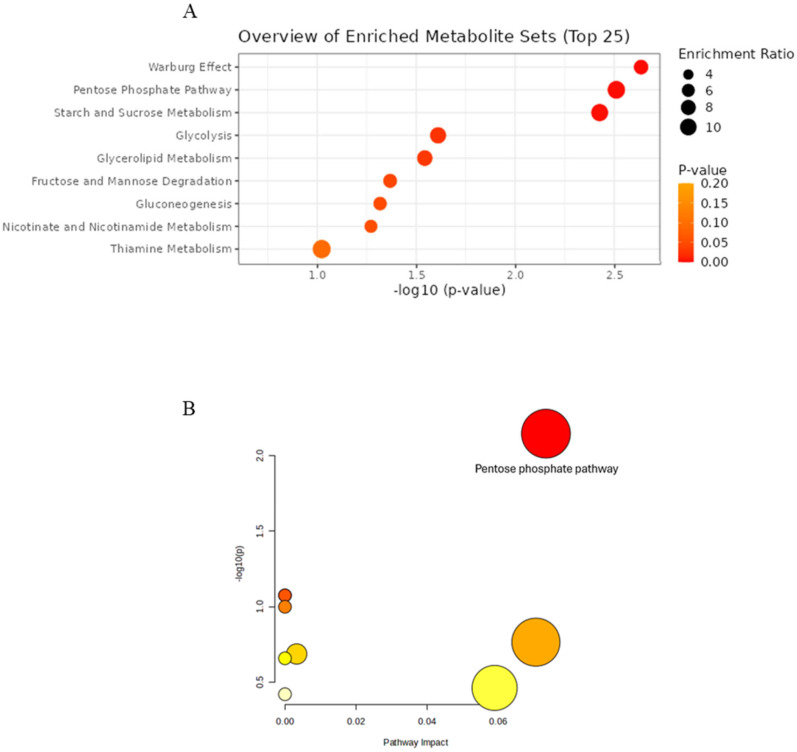
Analysis of impacted metabolic pathways of brain samples in HFHSD compared to HFHSD MOE group. (**A**) Enrichment pathway using SMPDB integrated into MetaboAnalyst 6.0. (**B**) Pathway analysis using KEGG pathways of *Rattus norvegicus*.

The most impacted pathways were the pentose phosphate pathway and starch and sucrose metabolism (*p* = 2.31 × 10^−3^ and *p* = 3.08 × 10^−3^, respectively), each with three overlapping metabolites. Additionally, four other related pathways were also considerably altered, each with two overlapping metabolites: glycolysis, glycerolipid metabolism, fructose and mannose degradation, and gluconeogenesis (*p* = 2.45 × 10^−2^, *p* = 2.86 × 10^−2^, *p* = 4.28 × 10^−2^ and *p* = 4.81 × 10^−2^, respectively) (Figure 9A). In addition, pathway analysis highlighted one pathway significantly impacted by MOE supplementation. The pentose phosphate pathway was found to be altered with two overlapping metabolites (*p* = 7.14 × 10^−2^, impact = 0.073) (Figure 9B).

Furthermore, metabolite expression profiles in brain samples were analyzed between the HFHSD MOE group and HFHSD group (Table 4).

Metabolite expression profiles of brain samples highlighted a consistent over-expression of all overlapping metabolites in the HFHSD MOE group in comparison to the HFHSD group when performing enrichment pathway analysis. Among the most impacted pathways were the pentose phosphate pathway with three hits (D-sedoheptulose 7-phosphate, D-erythrose 4-phosphate, and phosphate) and starch and sucrose metabolism, also with three hits (3-phosphoglyceric acid, phosphate, and beta-D-fructose 6-phosphate). These pathways were followed by glycolysis and glycerolipid metabolism, which shared two overlapping metabolites (3-Phosphoglyceric acid and Phosphate). Moreover, fructose/mannose degradation and gluconeogenesis were impacted, with two hits each and phosphate as a shared overlapped metabolite (GDP-L-fucose, 3-Phosphoglyceric acid, and phosphate, respectively) (Table 4).

Finally, pathway analysis based on Rattus norvegicus KEGG pathways showed only one significantly impacted pathway, the pentose phosphate pathway with also two overlapped metabolites (sedoheptulose 7-phosphate, D-Erythrose 4-phosphate), corroborating the enrichment analysis results (same as Table 4).

## 3. Discussion

Plant extracts offer a major advantage due to their rich content of bioactive compounds, capable of acting through multiple biochemical pathways to produce a range of physiological effects. In ethnoveterinary practice, *Melissa officinalis* has been widely used for its various effects, including anxiolytic activity [6]. In this study, the effects of *Melissa officinalis* were explored in an HFHSD model of rats using a non-targeted metabolomic approach. This study was a follow-up to a previous study that was carried out to investigate *Melissa officinalis’* anxiolytic effects in dogs, revealing an improvement in dogs’ behavior after MOE supplementation. Accordingly, the dosage selected for the present study was based on these prior findings [10]. This dose is further supported by the existing literature, which consistently employs comparable levels to evaluate physiological and behavioral effects of the extract in rodents [16,17]. In this study, a rat model was used allowing access to brain samples, especially the hypothalamus, to better understand the mechanisms underlying the anxiolytic effects of *Melissa officinalis*. Furthermore, the HFHSD model was used to investigate not only the anxiolytic effects of MOE but also its impact on obesity and glucose homeostasis. Therefore, non-targeted metabolomics as an exploratory approach was conducted to shed light on metabolic changes induced by MOE in HFHSD rats’ model, aiming to better understand MOE mechanisms of action, mainly related to anxiety as well as to obesity and glucose homeostasis.

This study was carried out on an HFHSD model with 45% fat. The results revealed that using this type of diet increased final body weight in rats to 611 g after 12 weeks. Based on a 2016 study classifying degrees of adiposity in rats, this weight corresponds to the obese category. However, the body weight alone can be a misleading indicator of obesity, and should be combined with other indicators, such as the adiposity index [18]. The HFHSD rats from this study exhibited an adiposity index of approximately 6%, which Leopoldo et al. categorized as overweight [18]. Taken together with previous reports, these results support the robustness of the obesity model applied in this study.

The findings of this study indicate that MOE slightly affected the body weight of HFHSD rats but the change was not statistically significant. Although several studies reported a significant reduction in body weight following supplementation with *Melissa officinalis* extracts, those studies employed different experimental animal models than the model with Wistar rats used in this study [9,19,20,21]. Specifically, some studies used mouse models [9,21], and mice are known to have different energy metabolisms compared to rats [22], potentially making them more prone to losing weight than rats. Other studies used Otsuka Long-Evans Tokushima fatty (OLETF) rats [20], a polygenic model programmed to develop obesity accompanied by several metabolic dysregulations [23]. While OLETF rats are genetically stable as an obesity model and tend to be more resistant to weight loss, a study investigating the effect of a *Melissa officinalis* extract on OLETF rats administrated a higher dose of 8000 mg/kg per feed, which may explain the observed weight reduction [20]. Furthermore, Wistar rats with diet-induced obesity display greater variability in response to an HFHSD, potentially making it more difficult to detect significant effects at lower doses of MOE.

Additionally, the anxiolytic effects of *Melissa officinalis* have been widely documented [6,24]. In this study, an EPM test was used to evaluate the anxiolytic effect of the hydro-alcoholic MOE in HFHSD rats. The results demonstrated that MOE did not improve the anxiety in HFHSD rats. In accordance, a recent study comparing the effects of an aqueous and hydro-alcoholic extract of *Melissa officinalis*, showed that the hydro-alcoholic extract did not reduce, using EPM, anxiety in a C57BL/6 mouse model. Notably, this study did not provide detailed information on extracts’ composition, limiting mechanistic interpretation [17]. In contrast, the hydro-alcoholic MOE used in the present study was characterized for its major bioactive compounds, with rosmarinic acid as the primary bioactive compound [10]. Furthermore, rosmarinic acid has been reported in the literature to exert anxiolytic activity [25]. While aqueous extracts may contain relatively higher proportions of rosmarinic acid [26], hydro-alcoholic extraction allows the recovery of a broader range of compounds, which may influence the observed effects. Differences in extraction solvent and extract composition may therefore contribute to the variability of reported anxiolytic effects. Moreover, in a previous study conducted by our team in dogs, MOE demonstrated a clear anxiolytic effect when assessed using a standardized evaluation grid [13]. However, although bioactive constituents previously associated with anxiolytic effects were present in the extract, their presence was not sufficient to induce anxiolytic-like effects in the present obese rat model. Furthermore, the 200 mg/kg dose of MOE was selected in this study in an exploratory manner, based on its previously demonstrated efficacy in our canine model [10]. Although this dose was sufficient to induce metabolic responses in the HFHSD rats, it did not produce anxiolytic effects in the EPM test. We acknowledge that applying an interspecies scaling strategy, such as the Human Equivalent Dose (HED) approach, would likely have led to a higher dose for rats to achieve effects comparable to those observed in dogs. The absence of anxiolytic effects may therefore be attributed to this dosing gap rather than to a lack of extract activity. However, the fact that no HED-based adjustment was performed does not affect the overall validity of our findings. On the contrary, it emphasizes the functional limits of MOE across species and provides a solid foundation for future dose–response studies to better define its anxiolytic potential.

Therefore, these results highlight the need for additional investigations to confirm and further explore MOE’s anxiolytic properties across different experimental models.

*Melissa officinalis* extracts are also known to regulate blood glucose levels [19]. In this study, OGTT results showed a reduction in blood glucose levels in HFHSD rats supplemented with MOE, although this decrease was not statistically significant at individual time points except at T0. However, the AUC revealed a significant overall decrease compared with HFHSD controls. These findings suggest that the MOE altered the overall glycemic response, despite the absence of changes at specific time-point measurements. The AUC of glucose measurements is considered a more reliable and sensitive indicator of glucose tolerance, providing a comprehensive assessment of glucose metabolism [27]. This approach allows for capturing the entire glycemic response over time, which may not be apparent at isolated time intervals, thereby explaining the results obtained in this study.

Non-targeted metabolomics, as an exploratory approach, has proven to be particularly relevant in cases where conventional or targeted measurements fail to detect differences, enabling the discovery of novel biomarkers [28]. To this end, untargeted metabolomics was carried out on plasma and hypothalamus samples of SD, HFHSD, and HFHSD rats supplemented with MOE. The analysis of plasma extracts indicated that the HFHSD in rats affected several pathways in comparison to SD rats, mainly bile acid (BA) synthesis and taurine and hypotaurine metabolism. In accordance, a study examining the effects of obesity on BA in serum samples found an increase in total BA levels, particularly conjugated BAs, in obese subjects compared to non-obese subjects [29]. An increase in BA biosynthesis during obesity is expected as BAs act as emulsifiers to facilitate dietary fat absorption. Beyond this classical role, BAs also serve as signaling molecules, involved in the regulation of lipid, glucose, and energy metabolisms [30,31]. The HFHSD in rats also impacted lipid metabolism, specifically of sphingolipids and glycerophospholipids. Specifically, an elevated level of sphingosine-1-phosphate (S1P) was observed in HFHSD rats compared to the SD group. Similarly, a study reported increased plasma S1P levels in mouse models of obesity and insulin resistance [32]. Furthermore, the mutation of sphingosine kinase 1, the gene encoding the enzyme responsible for S1P biosynthesis, improved insulin resistance and reduced adipocyte hypertrophy and inflammation in adipose tissue [33], thereby explaining the observed increase in S1P during the high-fat high-sucrose diet. Glycerophospholipid metabolism was notably affected, with a significant reduction in lysophosphatidylcholine (Lyso PC (18:2/0:0)) observed in the HFHSD rats compared to the SD group. Altered levels of lysophosphatidylcholine are recognized as a hallmark of obesity, typically characterized by a decrease in Lyso PC species [34], observations that align with the results of this study. Certain lyso PC species are known to exhibit anti-inflammatory properties; their reduction may therefore be caused by the chronic inflammation state often associated with obesity [35].

Next, non-targeted metabolomics demonstrated several impacted pathways in the HFHSD supplemented with MOE compared to the HFHSD alone. Plasma samples analysis highlighted an impact on BA biosynthesis and glycerophospholipid and pyrimidine metabolisms after supplementation with MOE. The observed decrease in conjugated bile acids following MOE supplementation may contribute to its anti-obesity effects. Explicitly, conjugated bile acids influence adiposity, energy metabolism, and glucose regulation [36,37,38] and are linked to anxiety [39,40]. MOE’s anxiolytic effects may involve their modulation as our previous study found decreased taurine-conjugated bile acids after supplementation [10], aligning with a study reporting higher levels observed in fearful dogs [39]. Furthermore, the analysis of plasma samples following MOE supplementation impacted glycerophospholipid metabolism, especially of lysophospholipids. In accordance, several studies have highlighted the role of lysophosphatidic acid in obesity, insulin resistance, and neuropsychiatric disorders such as anxiety and depression [40]. These results, consistent with prior research, show the role of bile acid and lipid metabolism in glucose levels regulation, obesity, and anxiety-related behaviors.

To the best of our knowledge, there are currently no studies investigating the effects of *Melissa officinalis* on hypothalamic metabolome. Interestingly, in this study, MOE administration following an HFHSD in rats impacted several metabolic pathways linked to pentose phosphate (PPP) and energy metabolism pathways. Supplementation with MOE in rats fed an HFHSD reduced blood glucose levels. These results align with studies demonstrating the hypoglycemic effects of *Melissa officinalis* extracts [19], suggesting improved insulin sensitivity and enhanced cellular glucose uptake. In the brain, glucose is the primary source of energy, accounting for approximately 20% of the body’s total glucose consumption [41]. The improvement in glucose tolerance following MOE supplementation may therefore improve brain glucose uptake, including the hypothalamus, supporting ATP generation, neurotransmitter biosynthesis, oxidative stress management, and cognitive functions such as learning and memory [42]. Glucose enters the brain through transporters in the blood–brain barrier and is metabolized primarily through glycolysis [42], explaining the effects of MOE supplementation on this pathway. However, in HFHSD rats, MOE predominantly influenced the PPP, likely as an adaptative response to oxidative stress, since PPP activation generates NADPH + H^+^, a key antioxidant [41]. NADPH + H+ is an essential cofactor for glutathione reductase, responsible for reducing oxidized glutathione back to its active form. Glutathione is known to protect neurons from oxidative stress [43], suggesting an indirect modulation of neurotransmitters through antioxidant defense. Interestingly, during depression, increased brain oxidative stress also boosts PPP activity to counteract ROS overproduction [44]. Furthermore, a 2021 study in in Drosophila revealed that neuronal glucose uptake during long-term memory formation supplies the PPP, underscoring its key role in memory [41]. These findings, together with our results, highlight the role of PPP in glucose homeostasis, obesity, and psychological disorders.

Assessing MOE’s effects on both plasma and hypothalamic metabolomes allow us to integrate systemic and central metabolic responses in the discussion. The results revealed that supplementation with MOE primarily affected BA metabolism in plasma and PPP in the hypothalamus. In the literature, BA receptors, including Faresoid X receptor (FXR) and Takeda G protein-coupled receptor 5 (TGR5), play key roles in energy metabolism [37,45,46]. In the brain, TGR5 is implicated in obesity and depression [47,48]. While MOE has not been shown to directly activate TGR5, it modules the gut microbiota in high-fat diet rats [49]. In this study, MOE decreased taurine-conjugated BAs, possibly via microbiota-mediated deconjugation [50], potentially raising taurine levels and unconjugated bile acids [50]. In the literature, unconjugated BAs can cross the blood–brain barrier (BBB) [37] and activate TGR5 [50]. Interestingly, TGR5 agonist administration in high-fat diet mice reduces obesity markers and regulated energy balance while hypothalamic TGR5 activation mitigates diet-induced obesity and its downregulation exacerbates obesity [47]. Furthermore, recent metabolomic studies have shown that TGR5 agonists affect BA biosynthesis and PPP, consistent with our results [51]. Beyond anti-obesity and glucose effects, the TGR5 agonist, INT-777, normalized memory deficits and anxiety-like behaviors in rats [52]. Furthermore, TGR5-deficient mice show anxiety and depression-like behaviors [48]. Collectively, our findings suggest that MOE modulates primarily bile acid metabolism in plasma and the pentose phosphate pathway in the hypothalamus. Alongside the existing literature, these results suggest a complex, multi-faceted MOE mechanism of action where bile acid signaling and hypothalamic energy metabolism collectively regulate obesity, glucose metabolism, and anxiety-like behaviors. As both experimental groups were exposed to the same HFHSD-induced obese conditions, the observed differences in pentose phosphate pathway and bile acids metabolism are more likely attributable to MOE supplementation rather than to the obese state itself. However, it remains unclear whether the pathways impacted by MOE are primarily related to its effects on obesity, glucose metabolism, or anxiety-related behaviors. Further studies are needed to validate the hypothesis regarding the proposed mechanism underlying MOE’s therapeutic effects.

## 4. Materials and Methods

### 4.1. Ethics

This present study was carried out in accordance with the European Union’s Directive 2010/63/EU governing the protection of animals used for scientific purposes. All procedures were compliant with these guidelines and received approval from the ethical committee of Pays de la Loire CEEA06 (APAFIS #44266-2022071814297107).

### 4.2. Animals and Experimental Design

Twenty-four male Wistar rats (purchased from Janvier Labs—22 Rte des Chênes Secs, 53,940 Le Genest-Saint-Isle) aged 6 weeks were used. The animals were adapted to the standard laboratory conditions for two weeks before the beginning of the experiments (12 h dark/light cycle). No rats were excluded from the study as all animals were clinically healthy, had had no prior experimental exposure, originated from the same breeding facility, and were housed under standardized conditions. These characteristics constituted the inclusion criteria of the study. Based on the literature, eight rats were used per group [17,53,54]. Rats were divided into three groups (*n* = 8) as follows: a control group maintained on a standard chow diet (SD, 18.50% protein, 54.2% carbohydrates, 4.5% fat, 3.15 kcal/g, 3430; Gravonit AG-Kliba Nafag, Kaiseraugst, Switzerland), a high-fat high-sucrose diet group (HFHSD, 20.17% protein, 34.67% carbohydrate, 45.19% fat, 4.69 kcal/g, D12451; Research Diets Inc., New Brunswick, NJ, USA) and an HFHSD group concurrently administered a hydro-alcoholic standardized Melissa officinalis extract (HFHSD MOE). *Melissa officinalis* extract (MOE) is a commercial extract prepared by hydro-alcoholic extraction of its leaves. MOE contains mainly hydroxycinnamic acids, with rosmarinic acid as the major compound, followed by chlorogenic acid and caffeic acid [10]. Over a period of 12 weeks, MOE was administrated via oral gavage at a dose of 200 mg/kg [10,16,17]. All the rats had ad libitum access to food and water. This was an open-label, non-randomized, and non-blinded study. At the end of the supplementation period, each rat was used to assess morphometric parameter measurements, elevated plus maze (EPM), and oral glucose tolerance test (OGTT).

### 4.3. Weight and Morphometrics Measurements

Rats’ body weight (BW) was monitored on a weekly basis. Body mass index (BMI), adiposity index (AI), and abdominal circumference (AC) were also measured after 12 weeks (W12). AC was measured in anesthetized rats placed in dorsal recumbency. A non-elastic measuring tape was passed underneath the animal and positioned horizontally around the abdomen at the level of maximal circumference. The tape was kept in contact with the skin without compressing the underlying soft tissues [55]. BMI was calculated as follows [55]:

BMI (g/cm^2^) = BW (g)/Naso-anal length^2^ (cm^2^)

AI was calculated by collecting visceral adipose tissue (VAT) after euthanasia [56]. VAT was blotted dry and weighed, allowing the calculation of AI as follows:

AI (%) = ΣVAT (g)/BW (g) × 100

### 4.4. Elevated Plus Maze Test (EPM)

Rats’ behavior was assessed using an EPM test. The EPM was set 60 cm above the floor and consisted of two open arms (36 × 6 cm) and 2 closed arms (36 × 6 × 15 cm). Each rat was individually placed in the maze’s center for a 5 min test period. To assess anxiety-like behaviors, the time spent and distances traveled in both open and closed arms were measured for each rat. All EPM experiments were conducted in a soundproofed, darkened room, with a single light source providing uniform illumination to the maze’s center. Data were recorded through a video tracking system and analyzed using BIOEPM-3C software.

### 4.5. Oral Glucose Tolerance Test (OGTT)

The effect of MOE on glucose homeostasis was assessed using the OGTT after 12 weeks of supplementation. Before the procedure, the rats were accustomed to the restraining tube for three consecutive days, for 15 min each day. A glucose solution was prepared by dissolving glucose in distilled water. Then, after a four-hour fasting period, the rats were administered glucose at a dose of 2 g/kg of body weight via oral gavage. Afterwards, blood samples were collected from the caudal vein at various time intervals (0, 15, 30, 45,60, 90, 120 min) and blood glucose levels (mg/dL) were monitored using a calibrated glucometer (StatStrip Xpress^®^2, Waltham, MA, USA).

### 4.6. Blood and Tissue Collection

At the end of the supplementation period, all rats were anesthetized with an intraperitoneal injection with ketamine-medetomidine (60 mg/kg ketamine and 120 µg/kg medetomidine). The anesthesia was verified by the disappearance of the paw withdrawal reflex after pinching the interdigital spaces. Next, euthanasia was performed by exsanguination at the level of the abdominal aorta, permitting the collection of almost 10 mL of blood and organs. The brain and visceral adipose tissue were collected. To perform the metabolomic analysis, plasma was isolated by blood centrifugation and hypothalamic tissue was dissected from the brain.

### 4.7. Statistical Analysis

The data were expressed as means ± standard error of means (SEMs). The distribution of the data was assessed for normality using the Shapiro–Wilk test. Inter-group comparisons were performed using one-way and two-way analysis of variance (ANOVA), as appropriate for the experimental design, followed by Tukey’s post hoc test for multiple comparisons. The probability value of *p* < 0.05 was considered statistically significant.

### 4.8. Metabolomic Analysis

#### 4.8.1. Metabolite Extraction from Plasma

Metabolites were extracted from plasma samples using a modified protocol based on Zhang et al., 2019 [57]. The extraction began with mixing 100 µL of plasma with 2 µL of proteinase K (20 mg/mL) and 2 µL of CaCl2 solution (250 mmol/L). The mixture was then incubated at 37 °C in a water bath for 15 min. Following incubation, protein precipitation was performed by adding 300 µL of chloroform/methanol solution (3:1, *v*/*v*). The solution was vortexed and incubated at −20 °C overnight, followed by centrifugation to collect the supernatant. The supernatant was then evaporated using GenevacTM EZ-2 evaporator (Genvatech Ltd., UK) and resuspended in dichloromethane/methanol (1:1, *v*/*v*) to obtain hydrophobic extracts (HPO) of plasma. A second extraction step was performed to the remaining precipitate by adding 300 µL of methanol/water (3:1, *v*/*v*). The mixture was then centrifuged and the resulting supernatant was collected. This second supernatant was evaporated and subsequently resuspended in acetonitrile/water (1:1, *v*/*v*) to obtain hydrophilic extracts (HPI) of plasma.

#### 4.8.2. Metabolite Extraction from Hypothalamus

Hypothalamic tissue samples were disrupted using the Bioprep 24-homogenizer (Allsheng Instruments Co., Ltd., Hangzhou, China) with 3 mm metallic beads. Samples were homogenized at 6.00 m/s for 40 s. Next, 2 µL of proteinase K (20 mg/mL) and 2 µL of CaCl_2_ solution (250 mmol/L) were added and samples were vortexed until complete homogenization was achieved. The remaining extraction steps for hypothalamic tissues followed the same protocol as that used for plasma. Consequently, each hypothalamic tissue sample yielded two distinct extracts: a hydrophobic hypothalamus extract and a hydrophilic hypothalamus extract.

#### 4.8.3. UHPLC-MS/MS Metabolomics Analysis

The analysis was carried out using ultra-high-performance liquid chromatography (UHPLC) ACQUITY H-Class PLUS (Waters, Manchester, UK) coupled to a quadrupole time-of-flight–high-resolution mass spectrometry (QTOF-HRMS) Xevo G2-XS (Waters, Manchester, UK) device. Chromatographic separation was performed using an HSS T3 column, 2.1 mm × 100 mm, with 1.8 µm particle size purchased from Waters. This column was used for plasma and hypothalamic samples due to its capability to separate both polar and apolar compounds thanks to its trifunctionally bonded C18 (T3) technology. Mobile phases A and B were water with 0.1% (*v*/*v*) formic acid and acetonitrile with 0.1% (*v*/*v*) formic acid, respectively. For plasma extracts, the following elution conditions were programmed as follows: (0–1 min) 2%B; (1–9 min) 2–35%B; (9–17 min) 35–100% with an elution rate of 0.4 mL/min. For hydrophobic hypothalamus extract, the elution conditions were carried out as follows: (0–1 min) 2% B; (1–3 min) 2–30% B; (3–5 min) 30–40% B; (5–10 min) 40–50% B; (10–13 min) 50–80% B; (13–15 min) 80–98% B. For hydrophilic hypothalamus extract, the elution conditions were modified as follows: (0–1 min) 2% B; (1–3 min) 2–50% B; (3–5 min) 50–60% B; (5–10 min) 60–70% B; (10–13 min) 70–80% B; (13–15 min) 80–98% B. Mass spectrometry analysis was conducted using negative ionization mode. The following conditions were established—capillary voltage: 0.5 kV; sampling cone: 40 kV; source offset: 80; source: 120 °C; desolvation: 500 °C; desolvation gas: 1000 L/h; cone gas: 100 L/h. Data was recorded using MS^E^.

#### 4.8.4. Data Treatment

MassLynx software was used for data acquisition. Subsequent data processing, including alignment, normalization, peak picking, and deconvolution, was conducted using Progenesis^®^ QI software (v3.0, Waters, Manchester, UK). Normalization and alignment were performed using quality control (QC) samples. Each QC was prepared as a pooled mixture of all study samples, separately for hydrophilic and hydrophobic samples obtained from the extraction procedure. During Q-TOF analysis, QC samples were injected multiple times as analytical replicates. Data processing was performed using Progenesis QI software, which selected the QC replicate that best represented all samples. This QC replicate was then used for normalization and alignment, ensuring consistent and reliable metabolite measurements across the dataset (Figures S2–S4).

To elucidate group differences, orthogonal partial least squares–discriminant analysis (OPLS-DA) was applied to the processed data. The reliability of the OPLS-DA results was validated through permutation tests implemented in R studio using the Ropls package [58]. A variable importance of projection (VIP) > 1 and *p*-value < 0.05 were applied to select features significantly different between the treated groups. Identification was performed using Human Metabolome database (HMDB) (https://hmdb.ca/, accessed on 10 November 2025) and MassBank of North America (MoNA) (https://mona.fiehnlab.ucdavis.edu/, accessed on 10 November 2025), integrated into Progenesis^®^ QI. The following identification criteria were applied to choose identifications: score > 35, mass error (ppm) < 5, and isotope similarity > 70. Investigation of enriched pathways was carried out using MetaboAnalyst 6.0 (https://www.metaboanalyst.ca/MetaboAnalyst/ModuleView.xhtml, accessed on 10 November 2025). The enrichment analysis was conducted using SMPDB (Small Molecule Pathway Database), which integrated pathway annotations based on normal human metabolic pathways. Additionally, pathway analysis was performed through MetaboAnalyst’s KEGG pathway module for *Rattus norvegicus*. Pathways were considered statistically significant when *p*-value < 0.05.

## 5. Conclusions

Building on a previous study investigating MOE’s impact on behavior and metabolomics [10], the present study provided deeper insight into its anxiolytic effects. Although MOE administration in HFHSD-fed rats did not result in statistically significant changes in body weight, blood glucose, or behavior, non-targeted metabolomics revealed the modulation of several metabolic pathways, mainly the bile acid and pentose phosphate pathways. These metabolic changes suggest a subsequent mechanism of action through which MOE may exert its multi-targeted effects on anxiety, obesity, and glucose homeostasis. Importantly, the paper underscores the value of untargeted metabolomics as a powerful tool to uncover potential mechanisms and biomarkers that may be overlooked by conventional analyses. These findings lay a foundation for future mechanistic studies and support the continued exploration of MOE as a promising plant-based additive with multi-faceted effects. While randomization and blinding were not implemented, the study provided meaningful preliminary insights into the effects of MOE. Furthermore, although there are physiological differences between rats and dogs, the rat is regarded in the literature as a relevant experimental model and has been used to study canine behavior [11,12]. Moreover, it should be emphasized that the use of an obese rat model represents a limitation as it introduces metabolic confounding factors and does not fully recapitulate the canine condition. Moreover, the metabolic pathways highlighted in this model may not reflect the same biological relevance in dogs, the target species of interest. The present results should therefore be interpreted as exploratory and hypothesis-generating rather than directly translatable. Future studies are warranted to confirm whether the same mechanisms are observed in dogs and to assess the consistency of these findings in the species of interest.

## Figures and Tables

**Table 1:** Metabolite names and their profile of pathway analysis in case of comparing HFHSD to SD group using KEGG pathways of *Rattus norvegicus* in plasma samples.

Pathway Name	Overlapping Metabolites	Metabolite Names	Profile	*p*-Value	Pathway Impact Score
Primary bile acid biosynthesis	4/46	Taurine	▲	5.50 × 10^−4^	0.098
		3a,7a,12a-Trihydroxy-5b-cholestan-26-al	▲		
		Glycocholic acid	▼		
		Taurocholic acid	▲		
Taurine and hypotaurine metabolism	2/8	Taurine	▲	2.06 × 10^−3^	0.428
		Taurocholic acid	▲		
Sphingolipid metabolism	3/32	Sphingosine 1-phosphate	▲	2.50 × 10^−3^	0.059
		Sphinganine 1-phosphate	▲		
		Ganglioside GM3 (d18:1/9Z-18:1)	▼		
Glycerophospholipid metabolism	2/36	LysoPC(18:2/0:0)	▼	4.02 × 10^−2^	0.122
		PE(14:1(9Z)/20:4(8Z,11Z,14Z,17Z))	▲		

▲ indicates an increase in metabolite levels in the HFHSD group compared with the SD group. ▼ indicates a decrease in metabolite levels in the HFHSD group compared with the SD group.

**Table 2:** Metabolite names and their profile of enrichment analysis in case of comparing HFHSD MOE to HFHSD group using SMPDB integrated into MetaboAnalyst in plasma samples.

Pathway Name	Overlapping Metabolites	Metabolite Names	Profile	*p*-Value
Bile acid biosynthesis	3/65	Taurocholic acid	▼	4.53 × 10^−3^
		Lithocholyltaurine	▼	
		Taurodeoxycholic acid	▼	

▼ indicates a decrease in metabolite levels in the HFHSD MOE group compared with the HFHSD group.

**Table 3:** Metabolite names and their profiles of pathway analysis in case of comparing HFHSD MOE to HFHSD group using KEGG pathways of *Rattus norvegicus* in plasma samples.

Pathway Name	Overlapping Metabolites	Metabolite Names	Profile	*p*-Value	Pathway Impact Score
Glycerophospholipid metabolism	2/36	LysoPE(0:0/18:1(11Z))	▲	1.71 × 10^−2^	0.156
		LysoPA(18:1(9Z)/0:0)	▲		
Pyrimidine metabolism	2/39	dTDP	▼	2.00 × 10^−2^	0.087
		Thymidine 3′,5′-cyclic monophosphate	▼		

▲ indicates an increase in metabolite levels in the HFHSD MOE group compared with the HFHSD group. ▼ indicates a decrease in metabolite levels in the HFHSD MOE group compared with the HFHSD group.

**Table 4:** Metabolite names and their profile of enrichment pathway analysis in case of comparing HFHSD to HFHSD MOE group using SMPDB integrated into MetaboAnalyst in brain samples.

Pathway Name	Overlapping Metabolites	Metabolite Names	Profile	*p*-Value
Pentose phosphate pathway	3/29	D-Sedoheptulose 7-phosphate	▲	3.08 × 10^−3^
		D-Erythrose 4-phosphate	▲	
		Phosphate	▲	
Starch and sucrose metabolism	3/31	3-Phosphoglyceric acid	▲	3.74 × 10^−3^
		Phosphate	▲	
		Beta-D-Fructose 6-phosphate	▲	
Glycolysis	2/23	3-Phosphoglyceric acid	▲	2.45 × 10^−2^
		Phosphate	▲	
Glycerolipid metabolism	2/25	3-Phosphoglyceric acid	▲	2.86 × 10^−2^
		Phosphate	▲	
Fructose and mannose degradation	2/31	GDP-L-fucose	▲	4.28 × 10^−2^
		Phosphate	▲	
Gluconeogenesis	2/33	3-Phosphoglyceric acid	▲	4.81 × 10^−2^
		Phosphate		

▲ indicates an increase in metabolite levels in the HFHSD MOE group compared with the HFHSD group.

## Data Availability

All data generated in this study are included in the Supplementary Materials.

## Supplementary Materials

The following supporting information can be downloaded at: https://www.mdpi.com/article/10.3390/ijms27052391/s1.

## Abbreviations

The following abbreviations have been used in this manuscript:

MOE	*Melissa officinalis* extract
SD	Standard diet
HFHSD	High-fat high-sucrose diet
HFHSD MOE	HFHSD supplemented with MOE
EPM	Elevated plus maze
OGTT	Oral glucose tolerance test
AUC	Area under the curve
GABA	γ-aminobutyric acid
GABA-T	γ-aminobutyric acid transaminase
ONIRIS	Nantes-Atlantic National College of Veterinary Medicine, Food Science and Engineering
BW	Body weight
BMI	Body mass index
AI	Adiposity index
VAT	Visceral adipose tissue
AC	Abdominal circumference
SEM	Standard error of the mean
HPO	Hydrophobic extract
HPI	Hydrophilic extract
ESI	Electrospray ionization
OPLS-DA	Orthogonal partial least squares–discriminant analysis
VIP	Variable importance in projection
KEGG	Kyoto Encyclopedia of Genes and Genomes
SMPDB	Small Molecule Pathway Database
UHPLC	Ultra-high-performance liquid chromatography
QTOF-HRMS	Quadrupole time-of-flight-high resolution mass spectrometry
HMDB	Human Metabolome Database
MoNA	MassBank of North America
OLETF	Otsuka Long-Evans Tokushima fatty
PPP	Pentose phosphate pathway
BA	Bile acids
FXR	Faresoid X receptor
TGR5	Takeda G protein-coupled receptor 5
