# Supplementary material for: Plasma and Brain Metabolomics Uncover Modulation of Bile Acid and Pentose Phosphate Pathways by Melissa officinalis in Obese Rat Model
Source: Int J Mol Sci. 2026 Mar 4;27(5):2391. doi: 10.3390/ijms27052391 (PMC12985921; doi:10.3390/ijms27052391)
Supplement: Supplementary file 1 [file ijms-27-02391-s001.zip › ijms-4149378-supplementary.pdf]

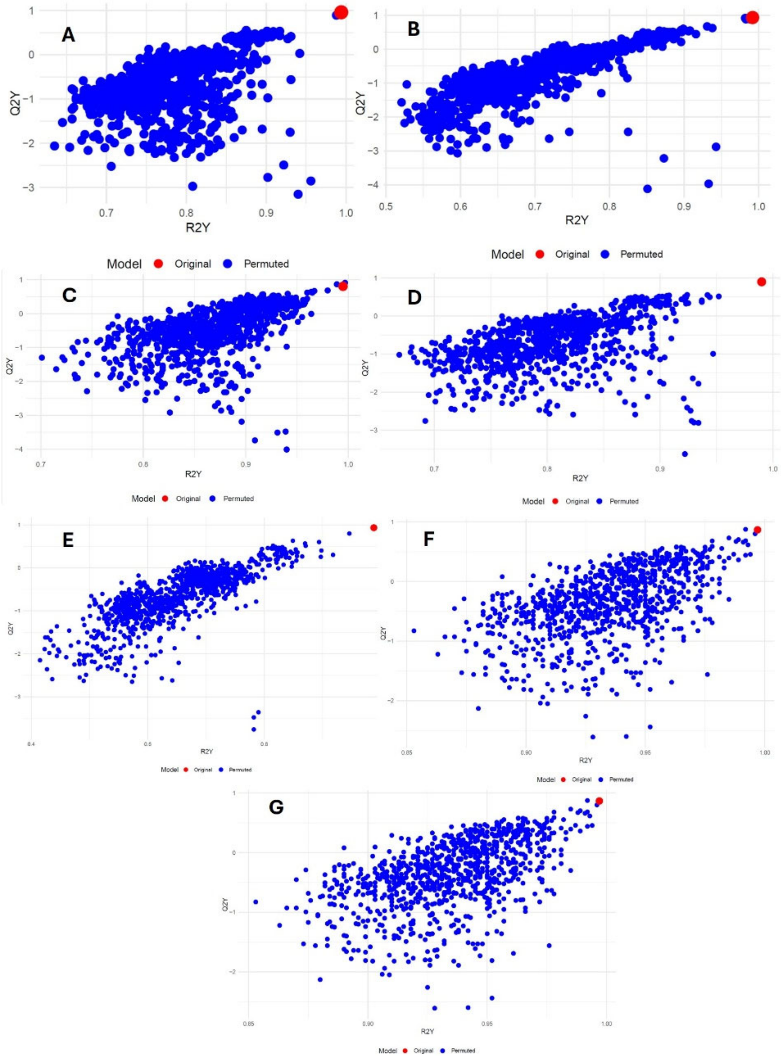

Figure S1: Permutations tests concerning plasma and brain samples. (A) HPO extracts of plasma samples in ESI^- (SD vs HFHSD). (B) HPI extracts of plasma samples in ESI^- (SD vs HFHSD). (C) HPO extracts of brain samples in ESI^- (SD vs HFHSD). (D) HPO extracts of plasma samples in ESI^- (HFHSD vs HFHSD MOE). (E) HPI extracts of plasma samples in ESI^- (HFHSD vs HFHSD MOE). (F) HPO extracts of brain samples in ESI^- (HFHSD vs HFHSD MOE). (G) HPI extracts of brain samples in ESI^- (HFHSD vs HFHSD MOE).

Table S1: Permutations test parameters concerning plasma and brain samples.

Plasma	SD vs HFHSD	HPO in ESI^-	R2Y= 0.994. Q2Y=0.960
	SD vs HFHSD	HPI in ESI^-	R2Y=0.992. Q2Y=0.934
	HFHSD vs HFHSD MOE	HPO in ESI^-	R2Y= 0.990. Q2Y=0.899
	HFHSD vs HFHSD MOE	HPI in ESI^-	R2Y=0.988. Q2Y=0.935
Brain	SD vs HFHSD	HPO in ESI^-	R2Y=0.995. Q2Y=0.805
	HFHSD vs HFHSD MOE	HPO in ESI^-	R2Y=0.991. Q2Y=0.879

	HFHSD vs HFHSD MOE	HPI in ESI$^-$	R2Y=0.997. Q2Y=0.867

Table S2: List of accepted identifications between control (SD) and HFHSD groups of HPO in ESI$^-$ of plasma samples.

m/z	RT (min)	HMDB ID	Accepted Description			
538,8125	0,5	HMDB0059921	Trimetaphosphoric acid	37,5	1,33	▲
231,0617	0,6	HMDB0258248	serine glutamate	48,3	-2,28	▲
166,0188	0,65	HMDB0014496	Thioguanine	38,1	-2,92	▲
290,0509	0,65	HMDB0252772	Glucose aspartate	42,4	-2,91	▲
130,0517	0,69	HMDB0002099	6-Methyladenine	38,2	-3,86	▼
262,105	0,71	HMDB0240254	Creatine riboside	47,2	2	▲
189,0887	0,72	HMDB0039222	N-(gamma-Glutamyl)ethanolamine	46,3	3,42	▼
272,1729	0,78	HMDB0028722	Arginylvaline	48,7	0,26	▼
262,0875	1,26	HMDB0028737	Asparaginyl-Methionine	39,5	3,13	▼
301,1528	1,5	HMDB0013016	Neuromedin B (1-3)	46,3	3,39	▲
187,1095	1,65	HMDB0000206	N6-Acetyl-L-lysine	43,1	3,75	▼
401,2101	1,93	HMDB0003128	Cortolone	40,1	0,17	▼
128,036	1,98	HMDB0006556	L-4-Hydroxyglutamate semialdehyde	38,9	4,96	▲
167,0218	2,03	HMDB0000289	Uric acid	44	4,5	▲
296,9807	2,03	HMDB0029219	Urolithin D	39	-0,15	▲
187,1095	2,2	HMDB0000759	Glycylleucine	46,9	3,87	▼
531,2418	2,21	HMDB0029015	Prolyl-Glutamine	40,2	-0,39	▲
173,0937	2,46	HMDB0028854	Glycylvaline	42,7	3,09	▼
267,0992	2,68	HMDB0028848	Glycyl-Phenylalanine	39,1	2,51	▼
231,1356	2,85	HMDB0013075	Spermic acid 2	39,9	2,34	▼
656,3924	3,19	HMDB0012342	PS(14:1(9Z)/14:1(9Z))	50,6	-1,3	▲
371,1936	3,28	HMDB0028695	Alanylproline	44,8	-0,04	▲
458,244	3,29	HMDB0114759	LysoPA(20:3(8Z,11Z,14Z)/0:0)	44,4	-1,91	▼
545,2835	3,43	HMDB0247808	Butyloxycarbonyl-leucyl-glycyl-arginine-4-nitroanilide	42,6	-1,11	▼
400,2342	3,48	HMDB0241254	2-Hydroxydodeca-4,6-dienoylcarnitine	40,6	0,24	▼
562,2385	3,52	HMDB0250586	L-Prolinamide, N-methyl-L-alanyl-(2S)-2-cyclohexylglycyl-N-(2-(2-oxazolyl)-4-phenyl-5-thiazolyl)-	41,9	1,3	▼
357,2243	3,55	HMDB0012983	Kinetensin 1-3	38,8	-3,63	▼
955,3744	3,6	HMDB0273193	PGP(18:3(9Z,12Z,15Z)/20:5(7Z,9Z,11E,13E,17Z)-3OH(5,6,15))	44,1	-4,09	▼
849,3715	3,62	HMDB0274883	PGP(i-12:0/20:3(6,8,11)-OH(5))	53,3	-1,45	▼
880,4182	3,62	HMDB0282835	PS(20:5(5Z,8Z,11Z,14Z,17Z)/20:5(5Z,8Z,11Z,14Z,16E)-OH(18))	54,8	1,09	▼
360,1785	3,65	HMDB0011627	Farnesylcysteine	41,3	4,75	▼
395,1587	3,69	HMDB0028720	Arginyltryptophan	46,5	-4,75	▼
361,1735	3,91	HMDB0060574	Perindoprilat	43,7	-2,91	▼
956,4398	3,96	HMDB0275027	PGP(i-14:0/LTE4)	51,8	3,42	▼
974,469	4	HMDB0116074	CDP-DG(a-13:0/i-18:0)	48	1,02	▼
1047,4602	4,02	HMDB0279446	PIP(PGF1alpha/18:3(9Z,12Z,15Z))	53,6	-1,74	▼

799,4664	4,08	HMDB0281079	PS(20:4(6E,8Z,11Z,13E)-2OH(5S,15S)/15:0)	50	1,62	▲
405,1881	4,1	HMDB0012643	20-Trihydroxy-leukotriene-B4	50,9	-3,53	▼
1006,4647	4,15	HMDB0247699	Pglu-Leu-Thr-Phe-Thr-Ser-Ser-Trp-GlyNH2	50,7	0,72	▼
278,1149	4,22	HMDB0028738	Asparaginyl-Phenylalanine	41,1	0,91	▼
446,2265	4,22	HMDB0252877	Glycyl-histidyl-arginyl-proline	50,5	-0,98	▼
746,4136	4,24	HMDB0008872	PE(14:1(9Z)/20:4(8Z,11Z,14Z,17Z))	51	-4,61	▲
1078,4972	4,24	HMDB0293747	CDP-DG(20:4(6E,8Z,11Z,13E)-2OH(5S,15S)/i-16:0)	48,5	-4,93	▼
229,0036	4,29	HMDB0245324	2-Thiouric acid	39,5	-0,74	▲
871,3992	4,36	HMDB0274859	PGP(i-12:0/20:4(8Z,11Z,14Z,17Z)-2OH(5S,6R))	45,9	-2,85	▲
420,056	4,37	HMDB0000912	Succinyladenosine	36,1	-0,75	▼
992,5025	4,39	HMDB0290819	CDP-DG(16:0/18:1(12Z)-O(9S,10R))	50,7	0,61	▼
1128,4917	4,41	HMDB0280630	PIP(LTE4/22:5(4Z,7Z,10Z,13Z,16Z))	49,4	2,35	▼
920,4461	4,43	HMDB0116052	CDP-DG(18:2(9Z,11Z)/i-12:0)	44,1	1,84	▼
611,313	4,43	HMDB0266643	PA(8:0/18:1(12Z)-O(9S,10R))	42,6	1,57	▼
939,3849	4,48	HMDB0273052	PGP(PGJ2/18:3(6Z,9Z,12Z))	42,8	1,88	▼
456,2949	4,51	HMDB0241486	(6,12)-11-Hydroxyhexadecadienoylcarnitine	50,3	-4,25	▲
901,4755	4,53	HMDB0276265	PI(6 keto-PGF1alpha/16:2(9Z,12Z))	52,4	3,83	▼
616,3277	4,65	HMDB0288875	PC(20:4(6E,8Z,11Z,13E)-2OH(5S,15S)/2:0)	48,9	3,41	▼
1047,4387	4,66	HMDB0242732	Vasotocin	56,6	-0,71	▼
963,4647	4,66	HMDB0293522	CDP-DG(i-14:0/18:1(12Z)-O(9S,10R))	51	0,9	▼
983,4708	4,7	HMDB0281976	PS(LTE4/18:4(6Z,9Z,12Z,15Z))	52,5	-0,2	▼
472,2471	4,71	HMDB0011510	LysoPE(18:4(6Z,9Z,12Z,15Z)/0:0)	42,9	0,37	▼
300,1939	4,71	HMDB0028945	Lysylarginine	48,4	4,57	▼
812,3201	4,71	HMDB0251612	Dpp-II	45,1	4,54	▼
961,4613	4,73	HMDB0281872	PS(18:3(9Z,12Z,15Z)/LTE4)	50	-1,88	▼
650,294	4,84	HMDB0250699	Cys-Arg-Glu-Lys-Ala	50,5	0,5	▼
528,2975	4,85	HMDB0241822	(5Z)-7-[(1R,2R,3R)-3-Hydroxy-2-[(1E,3S)-3-hydroxy-5-phenylpent-1-en-1-yl]-5-oxocyclopentyl]hept-5-enoylcarnitine	50,7	1,62	▼
656,3906	4,89	HMDB0012342	PS(14:1(9Z)/14:1(9Z))	51,1	-4,02	▼
891,4694	4,91	HMDB0276788	PI(PGJ2/18:3(6Z,9Z,12Z))	55,8	3,15	▲
1077,5078	4,91	HMDB0279757	PIP(PGF1alpha/20:2(11Z,14Z))	53,6	-1,03	▼
1187,6229	4,96	HMDB0259343	Tyr-ile-gly-ser-arg	42,9	4,28	▼
970,455	4,97	HMDB0274404	PGP(LTE4/a-15:0)	43,9	2,93	▼
783,4081	5,03	HMDB0264348	PA(18:4(6Z,9Z,12Z,15Z)/5-iso PGF2VI)	49,4	-1,16	▼
842,4621	5,04	HMDB0282834	PS(20:5(5Z,8Z,11Z,14Z,16E)-OH(18R)/20:5(5Z,8Z,11Z,14Z,17Z))	53,8	0,89	▼
690,3572	5,12	HMDB0059792	Opiorphin	51,4	-0,74	▼
1152,5416	5,26	HMDB0280213	PIP(22:2(13Z,16Z)/LTE4)	52,9	-4,25	▼

348,1937	5,32	HMDB0029225	Coutaric acid	43,8	2,47	▼
743,3928	5,33	HMDB0264375	PA(20:5(7Z,9Z,11E,13E,17Z)-3OH(5,6,15)/18:4(6Z,9Z,12Z,15Z))	53,7	-0,22	▲
1023,5842	5,42	HMDB0284476	PE(24:0/LTE4)	49,3	-3,86	▼
540,3013	5,44	HMDB0242415	Deoxycholylcysteine	39,5	2,57	▼
995,4531	5,49	HMDB0278631	PIP(16:2(9Z,12Z)/20:4(5Z,8Z,11Z,14Z)-OH(19S))	47,5	-0,96	▼
356,2565	5,54	HMDB0094648	Leu-Leu-Leu	40,2	2,82	▼
1120,5432	5,54	HMDB0293117	CDP-DG(a-21:0/20:5(6E,8Z,11Z,14Z,17Z)-OH(5))	48,7	1,82	▼
890,437	5,6	HMDB0112704	PS(20:5(5Z,8Z,11Z,14Z,17Z)/22:6(4Z,7Z,10Z,13Z,16Z,19Z))	50,7	-1,21	▲
705,3612	5,71	HMDB0262669	PA(10:0/PGD2)	46,6	-1,31	▼
1145,5096	5,71	HMDB0266577	PA(2:0/PGD1)	42	2,57	▼
775,4706	5,79	HMDB0263580	PA(20:3(8Z,11Z,14Z)-O(5,6)/18:0)	48,4	2,67	▲
1080,5262	5,88	HMDB0277206	PI(LTE4/20:2(11Z,14Z))	49,8	0,65	▼
661,3344	5,93	HMDB0260304	Myelopeptides	49,4	-1,58	▲
845,4388	6,05	HMDB0265174	PA(TXB2/20:4(5Z,8Z,11Z,14Z))	52,1	1,28	▼
613,2464	6,1	HMDB0006868	S-(2-Methylpropionyl)-dihydrolipoamide-E	37,2	-2,64	▼
850,4328	6,24	HMDB0264338	PA(18:4(6Z,9Z,12Z,15Z)/LTE4)	48,1	-0,7	▼
1152,5355	6,31	HMDB0293121	CDP-DG(a-21:0/20:5(7Z,9Z,11E,13E,17Z)-3OH(5,6,15))	46,3	3,96	▼
914,5224	6,48	HMDB0283257	PS(22:6(5Z,8E,10Z,13Z,15E,19Z)-2OH(7S, 17S)/22:4(7Z,10Z,13Z,16Z))	39,1	3,84	▲
321,0451	6,53	HMDB0029920	beta-D-3-Ribofuranosyluric acid	39,6	-0,48	▼
514,2843	7,11	HMDB0000036	Taurocholic acid	43,9	-0,2	▲
906,6568	7,2	HMDB0288654	PC(24:0/20:5(5Z,8Z,11Z,14Z,16E)-OH(18R))	35,1	-2,79	▲
307,066	7,34	HMDB0252779	Glucose lactate acetate	38,7	-3,39	▲
464,3015	7,48	HMDB0000138	Glycocholic acid	37,3	-0,48	▼
407,2797	8,16	HMDB0000326	1b,3a,12a-Trihydroxy-5b-cholanoic acid	43,2	-1,36	▼
524,277	9,17	HMDB0241619	(4Z,7Z,10Z,14E,16Z,19Z)-13-Hydroxydocosa-4,7,10,14,16,19-hexaenoylcarnitine	48,3	-2,79	▲
476,2787	9,18	HMDB0011477	LysoPE(0:0/18:2(9Z,12Z))	42,9	0,9	▼
552,3091	9,24	HMDB0011529	LysoPE(24:6(6Z,9Z,12Z,15Z,18Z,21Z)/0:0)	51,5	-0,76	▲
554,3029	9,25	HMDB0010386	LysoPC(18:2(9Z,12Z)/0:0)	41,1	1,97	▼
687,544	9,36	HMDB0240617	SM(d16:1/17:0)	44,1	-0,88	▲
742,5387	9,39	HMDB0009286	PE(20:2(11Z,14Z)/16:0)	49,7	-0,71	▲
799,5277	9,39	HMDB0265733	PA(22:6(4Z,8Z,10Z,13Z,16Z,19Z)-OH(7)/22:1(13Z))	49,3	-0,74	▲
737,5359	9,39	HMDB0296506	DG(PGJ2/0:0/21:0)	49,6	2,95	▲
540,3311	9,44	HMDB0010382	LysoPC(16:0/0:0)	49,2	0,89	▼
480,3097	9,44	HMDB0241520	9-Hydroxyoctadecanoylcarnitine	54,5	0,13	▼

452,2787	9,5	HMDB0296941	DG(2:0/0:0/20:3(8Z,11Z,14Z)-2OH(5,6))	50,4	-0,55	▲
802,5603	9,53	HMDB0009549	PE(22:2(13Z,16Z)/15:0)	47,2	-0,07	▲
912,5524	9,53	HMDB0284876	PE(DiMe(13,5)/20:4(5Z,8Z,10E,14Z)-OH(12S))	50,2	-0,37	▲
554,3464	10,11	HMDB0011511	LysoPE(20:0/0:0)	49,6	0,19	▼
745,5508	10,14	HMDB0240638	SM(d18:2(4E,14Z)/16:0)	46,4	1,02	▼
209,1551	10,32	HMDB0035881	11-Hydroxy-9-tridecenoic acid	42,8	1,54	▲
215,094	10,32	HMDB0250630	Cyclo(his-pro)	40,6	0,9	▲

Table S3: List of accepted identifications between control (SD) and HFHSD groups of HPI in ESI^- of plasma samples.

m/z	RT (min)	HMDB ID	Accepted Description	Score	ppm	Trend
225,1	0,6	HMDB0028878	Histidylalanine	45,7	2,47	▲
124,008	0,62	HMDB0000251	Taurine	38,6	4,33	▲
215,033	0,65	HMDB0249118	beta-D-galactosyl	41,8	3,28	▲
232,095	0,67	HMDB0029037	Serylglutamine	43,4	3,12	▼
346,139	0,71	HMDB0028711	Arginylhistidine	39,5	-3,74	▲
262,105	0,71	HMDB0028790	Glutaminylalanine	49,2	2,77	▲
244,167	0,71	HMDB0028964	Lysylvaline	45	1,7	▲
253,131	0,76	HMDB0005767	Homoanserine	41,2	1,62	▲
292,115	0,81	HMDB0011654	2-(3-Carboxy-3-(methylammonio)propyl)-L-histidine	48,8	0,23	▲
267,147	0,98	HMDB0028888	Histidylisoleucine	48,3	0,78	▼
235,076	0,98	HMDB0029045	Serylmethionine	44,6	2,72	▼
274,141	1	HMDB0029154	gamma-Glutamyllysine	55,8	-1,18	▼
414,248	1	HMDB0242076	N-Eicosapentaenoyl Methionine	48,5	1,41	▼
187,109	1,65	HMDB0000206	N6-Acetyl-L-lysine	42,3	1,86	▼
656,393	3,09	HMDB0012342	PS(14:1(9Z)/14:1(9Z))	51,3	-0,91	▲
304,152	3,1	HMDB0028952	Lysylhydroxyproline	53,5	1,27	▼
471,289	3,29	HMDB0003533	3a,7a,12a-Trihydroxy-5b-cholestan-26-al	47,2	2,34	▲
430,264	3,42	HMDB0241957	N-Oleoyl Cysteine	50,5	1,1	▼
618,341	3,58	HMDB0288899	PC(PGF1alpha/2:0)	49	-0,21	▼
501,296	3,6	HMDB0242012	N-Docosahexaenoyl Glutamine	47,1	-1,88	▼
397,134	3,65	HMDB0004448	17beta-Estradiol 3-sulfate	42	2,35	▼
755,389	3,69	HMDB0001038	Angiotensin IV	54,3	0,31	▼
360,178	3,74	HMDB0011627	Farnesylcysteine	51,9	4,32	▼
288,157	3,77	HMDB0028959	Lysylproline	52	3,26	▼
472,286	3,79	HMDB0242024	N-Docosahexaenoyl Tyrosine	48,8	0,99	▼
366,132	3,86	HMDB0241716	5-Hydroxyoct-2-enedioylcarnitine	49,8	-0,21	▼
1002,53	3,88	HMDB0269630	PG(LTE4/20:2(11Z,14Z))	46,8	-4,77	▲
344,183	3,89	HMDB0251528	DL-Proline	42,7	0,93	▼
687,364	4,02	HMDB0262800	PA(12:0/18:1(12Z)-2OH(9,10))	48,3	-0,98	▼

698,381	4,02	HMDB0262921	PA(20:5(7Z,9Z,11E,13E,17Z)-3OH(5,6,15)/13:0)	49,4	0,13	▼
926,492	4,03	HMDB0283260	PS(22:4(7Z,10Z,13Z,16Z)/20:4(6E,8Z,11Z,13E)-2OH(5S,15S))	49,9	-4,02	▼
800,464	4,08	HMDB0260859	PE(22:6(4Z,7Z,10Z,12E,16Z,19Z)-OH(14)/15:0)	53,1	-0,47	▲
728,358	4,08	HMDB0262677	PA(10:0/LTE4)	45,6	-2,77	▲
458,261	4,24	HMDB0013253	N-Acetylhistamine	40,9	-4,76	▲
272,162	4,24	HMDB0246805	5-Hydroxylysinonorleucine	53,2	2,1	▼
746,419	4,24	HMDB0264130	PA(PGJ2/18:3(6Z,9Z,12Z))	52,2	1,46	▲
281,115	4,25	HMDB0028799	Glutaminylhistidine	44,5	2,74	▲
689,398	4,34	HMDB0115029	PA(18:4(6Z,9Z,12Z,15Z)/15:0)	49	3,66	▲
573,319	4,34	HMDB0297138	DG(20:4(6Z,8E,10E,14Z)-2OH(5S,12R)/0:0/8:0)	52,2	-1,71	▼
993,507	4,39	HMDB0275643	PGP(6 keto-PGF1alpha/i-20:0)	53,9	-1,31	▼
1014,48	4,39	HMDB0293731	CDP-DG(18:1(12Z)-O(9S,10R)/i-16:0)	53,5	-2,3	▼
564,236	4,41	HMDB0266513	PA(2:0/6 keto-PGF1alpha)	45,7	1,05	▼
911,506	4,43	HMDB0269691	PG(20:2(11Z,14Z)/PGF1alpha)	54,1	-0,21	▼
920,445	4,44	HMDB0283533	PS(PGJ2/22:6(4Z,7Z,10Z,13Z,16Z,19Z))	56,7	-3,78	▼
440,152	4,48	HMDB0062779	Cortisol 21-sulfate	41,7	-0,84	▼
1154,57	4,53	HMDB0294322	CDP-DG(PGE2/i-22:0)	51,9	1,79	▼
1014,51	4,56	HMDB0279031	PIP(PGF1alpha/18:1(15Z))	51,8	-1,85	▼
371,231	4,59	HMDB0013327	Dodecanedioylcarnitine	44,6	-2,99	▼
761,384	4,63	HMDB0263023	PA(22:6(5Z,8E,10Z,13Z,15E,19Z)-2OH(7S, 17S)/14:0)	49,4	4,93	▼
417,172	4,66	HMDB0252497	Fructosylamine	41,3	-2,19	▼
799,381	4,68	HMDB0271090	PG(20:4(7E,9E,11Z,13E)-3OH(5S,6R,15S)/i-12:0)	53,7	0,14	▼
512,308	4,77	HMDB0242023	N-Docosahexaenoyl Tryptophan	47,3	4,32	▼
415,232	4,78	HMDB0252758	Glutamylleucylarginine	45,7	1,21	▲
1025,58	4,85	HMDB0277002	PI(TXB2/20:0)	52,5	1,87	▲
962,49	4,91	HMDB0010030	PIP(22:5(7Z,10Z,13Z,16Z,19Z)/16:0)	37,6	-3,95	▲
974,512	4,92	HMDB0278781	PIP(18:0/18:1(12Z)-2OH(9,10))	49,3	-2,68	▼
1022,48	4,92	HMDB0293561	CDP-DG(PGD1/i-14:0)	51,8	2,6	▲
596,3	4,96	HMDB0288835	PC(PGJ2/2:0)	47,3	1,13	▲
1058,58	4,97	HMDB0116204	CDP-DG(i-14:0/i-24:0)	53	-3,31	▲
360,195	4,97	HMDB0244860	1alpha-Hydroxycorticosterone	46,1	-0,07	▼
799,419	4,97	HMDB0274321	PGP(18:1(12Z)-2OH(9,10)/a-13:0)	46,7	2,78	▼
212,003	4,99	HMDB0000682	Indoxyl sulfate	40,1	1,31	▼
516,298	5,01	HMDB0241812	(4Z,7Z,10S,11E)-10-Hydroperoxy-12-[(1R,4S,5S,6R)-6-[(2Z)-pent-2-en-1-yl]-2,3-dioxabicyclo[2.2.1]heptan-5-yl]dodeca-4,7,11-trienoylcarnitine	46,4	2,16	▲
421,21	5,03	HMDB0032960	1-Octen-3-yl primeveroside	47,5	4,79	▼
753,415	5,09	HMDB0264373	PA(22:6(5Z,8E,10Z,13Z,15E,19Z)-2OH(7S, 17S)/18:4(6Z,9Z,12Z,15Z))	52,3	1,53	▼
837,373	5,12	HMDB0274324	PGP(a-13:0/18:1(9Z)-O(12,13))	49,3	-0,29	▼

569,304	5,14	HMDB0003577	VPGPR Enterostatin	48,9	-3,17	▲
915,49	5,14	HMDB0116451	PGP(20:1(11Z)/18:2(9Z,12Z))	47,9	-2,92	▲
820,457	5,18	HMDB0261447	PE(5-iso PGF2VI/18:2(9Z,12Z))	54,5	3,74	▼
437,203	5,21	HMDB0252827	Glycylphenylalanylleucylglycine	50,1	-3,52	▼
896,546	5,21	HMDB0261847	PE(6 keto-PGF1alpha/20:0)	51,6	3,85	▼
1063,51	5,21	HMDB0278348	PI(LTE4/22:6(4Z,7Z,10Z,13Z,16Z,19Z))	52,7	-4,38	▲
775,414	5,23	HMDB0271131	PG(i-12:0/22:6(4Z,7Z,10Z,13E,15E,19Z)-OH(17))	51,8	-3,59	▲
613,321	5,25	HMDB0002596	Deoxycholic acid 3-glucuronide	46,1	-2,69	▼
538,236	5,28	HMDB0011518	LysoPE(20:4(8Z,11Z,14Z,17Z)/0:0)	50,2	3,08	▼
348,194	5,32	HMDB0303977	(5Z)-(15S)-11-alpha-hydroxy-9,15-dioxoprosta-13-enoate	47,5	-1,83	▼
741,374	5,35	HMDB0262774	PA(12:0/6 keto-PGF1alpha)	55,8	-1,28	▼
698,408	5,37	HMDB0028960	Lysylserine	42,7	3,15	▼
544,21	5,38	HMDB0266556	PA(20:5(7Z,9Z,11E,13E,17Z)-3OH(5,6,15)/2:0)	47	2,27	▼
631,311	5,4	HMDB0033207	Coagulin R 3-glucoside	53,7	-2,59	▼
605,33	5,49	HMDB0013328	Pimelylcarnitine	44,2	0,66	▼
392,185	5,51	HMDB0032587	Pteroside Z	42	-1,33	▼
838,44	5,52	HMDB0262561	PE(20:4(5Z,8Z,11Z,14Z)-OH(16R)/20:5(5Z,8Z,11Z,14Z,17Z))	48,1	-3,52	▼
1064,56	5,54	HMDB0116059	CDP-DG(18:2(9Z,11Z)/i-19:0)	50,8	1,45	▲
890,441	5,6	HMDB0263923	PA(18:2(9Z,11Z)/LTE4)	52,8	-0,68	▲
900,493	5,62	HMDB0116457	PGP(20:3(8Z,11Z,14Z)/20:3(5Z,8Z,11Z))	43,6	-0,03	▼
782,404	5,62	HMDB0280888	PS(18:3(10,12,15)-OH(9)/14:0)	50,5	2,97	▼
1191,63	5,67	HMDB0293194	CDP-DG(TXB2/a-25:0)	53,1	-2,76	▲
599,371	5,73	HMDB0294555	DG(10:0/0:0/20:4(8Z,11Z,14Z,17Z)-2OH(5S,6R))	49,7	-1,07	▼
356,256	5,79	HMDB0094648	Leu-Leu-Leu	43,2	0,87	▼
775,47	5,79	HMDB0115373	PA(22:5(4Z,7Z,10Z,13Z,16Z)/22:6(4Z,7Z,10Z,13Z,16Z,19Z))	48,9	-0,48	▲
932,429	5,85	HMDB0282812	PS(TXB2/20:5(5Z,8Z,11Z,14Z,17Z))	46,6	-4,8	▼
1056,52	5,9	HMDB0279595	PIP(TXB2/20:1(11Z))	44,4	2,64	▼
841,464	5,93	HMDB0265887	PA(PGE2/22:4(7Z,10Z,13Z,16Z))	54	0,51	▼
555,346	5,93	HMDB0297082	DG(20:4(5Z,8Z,11Z,14Z)-OH(19S)/0:0/8:0)	39	-0,47	▼
613,362	6,04	HMDB0115608	PA(10:0/a-17:0)	43,1	-4,48	▼
845,439	6,05	HMDB0272401	PGP(20:4(5Z,8Z,11Z,14Z)-OH(20)/16:1(9Z))	50,1	2,11	▼
728,408	6,1	HMDB0264365	PA(20:5(5Z,8Z,11Z,14Z,16E)-OH(18R)/18:4(6Z,9Z,12Z,15Z))	42	0,86	▼
1090	6,17	HMDB0069208	TG(22:0/21:0/i-22:0)	35,4	2,37	▲
986,53	6,17	HMDB0116058	CDP-DG(18:2(9Z,11Z)/i-18:0)	48,8	2,61	▼
886,522	6,29	HMDB0284330	PE(22:5(7Z,10Z,13Z,16Z,19Z)/PGF1alpha)	55,6	0,79	▼
1185,68	6,33	HMDB0004843	Ganglioside GM3 (d18:1/9Z-18:1)	45,6	-3,1	▼
661,387	6,33	HMDB0263105	PA(20:4(6E,8Z,11Z,14Z)+=O(5)/14:1(9Z))	48	-1,22	▼
901,492	6,36	HMDB0276836	PI(22:6(4Z,7Z,10Z,12E,16Z,19Z)-OH(14)/18:3(6Z,9Z,12Z))	52,1	4,88	▼

999,497	6,4	HMDB0273947	PGP(PGF1alpha/22:4(7Z,10Z,13Z,16Z))	45,9	-0,8	▼
514,278	6,55	HMDB0241876	(5Z)-7-[(1R,2R,5S)-5-Hydroxy-2-[(1E,3S,5Z)-3-hydroxyocta-1,5-dien-1-yl]-3-oxocyclopentyl]hept-5-enoylcarnitine	47,1	-0,37	▲
1021,5	6,59	HMDB0273880	PGP(22:4(7Z,10Z,13Z,16Z)/PGF2alpha)	48,8	-2,03	▼
1060,53	6,61	HMDB0291002	CDP-DG(PGF2alpha/18:0)	52,4	4,61	▼
681,323	6,75	HMDB0251632	Tyr-D-thr-gly-phe-leu-thr	43,7	-2,81	▲
899,523	6,86	HMDB0266466	PA(22:6(4Z,7Z,11E,13Z,15E,19Z)-2OH(10S,17)/24:1(15Z))	39,2	2,54	▼
429,192	6,89	HMDB0251969	Estradiol-17beta-glucuronide	45,4	0,32	▲
407,279	8,17	HMDB0000326	1b,3a,12a-Trihydroxy-5b-cholanoic acid	43,9	-3,3	▼
378,242	8,65	HMDB0000277	Sphingosine 1-phosphate	45,6	-0,07	▲
380,257	8,81	HMDB0001383	Sphinganine 1-phosphate	43,4	-1,53	▲
524,279	9,18	HMDB0241623	(4Z,8Z,10Z,13Z,16Z,19Z)-7-Hydroxydocosa-4,8,10,13,16,19-hexaenoylcarnitine	44,8	0,23	▲
792,534	9,2	HMDB0008909	PE(15:0/22:2(13Z,16Z))	43,9	3,66	▲
588,331	9,27	HMDB0010395	LysoPC(20:4(5Z,8Z,11Z,14Z)/0:0)	51,3	0,61	▲
528,309	9,27	HMDB0241829	(10E)-11-(3,4-Dimethyl-5-pentylfuran-2-yl)undec-10-enoylcarnitine	45,2	-2,23	▲
391,282	9,29	HMDB0242127	Docosanedioic acid	39,9	-1,54	▼
656,318	9,29	HMDB0288833	PC(PGF2alpha/2:0)	44,9	-0,73	▲
540,332	9,44	HMDB0010382	LysoPC(16:0/0:0)	51,5	1,64	▲
480,31	9,44	HMDB0013154	12-Hydroxy-12-octadecanoylcarnitine	52,6	1,41	▲
452,279	9,51	HMDB0296941	DG(2:0/0:0/20:3(8Z,11Z,14Z)-2OH(5,6))	51,1	0,1	▲
544,268	9,58	HMDB0011489	LysoPE(0:0/20:5(5Z,8Z,11Z,14Z,17Z))	52,6	-0,02	▲
557,321	9,6	HMDB0242387	Cholylglutamine	55,1	-0,02	▲
540,331	9,6	HMDB0243971	1-Nonadecanoyl-glycero-3-phosphoethanolamine	55,8	0,95	▲
478,293	9,75	HMDB0253282	hydroxyoctadecenoylcarnitine	47,4	-1,33	▲
436,283	9,79	HMDB0011152	LysoPE(P-16:0/0:0)	50,4	-1,92	▲
581,325	9,79	HMDB0297216	DG(22:6(5Z,7Z,10Z,13Z,16Z,19Z)-OH(4)/8:0/0:0)	50,3	-0	▼
566,347	9,85	HMDB0010385	LysoPC(18:1(11Z)/0:0)	54,2	1,34	▲
506,325	9,85	HMDB0241573	(13Z)-3-Hydroxyicos-13-enoylcarnitine	56,7	-0,14	▲
462,299	9,99	HMDB0011130	LysoPE(18:0/0:0)	50,8	-0,95	▲
745,55	10,1	HMDB0240638	SM(d18:2(4E,14Z)/16:0)	50,9	-0,2	▼
599,317	10,4	HMDB0240261	LysoPI(18:0/0:0)	41,4	-4,87	▲
568,362	10,5	HMDB0010384	LysoPC(18:0/0:0)	42,9	0,2	▲
508,341	10,5	HMDB0012108	LysoPC(17:0/0:0)	54,2	-0,19	▲

Table S4: List of accepted identifications between control (HFHSD) and HFHSD MOE groups of HPO in ESI^{-} of plasma samples.

m/z	RT (min)	HMDB ID	Accepted Description	Score	ppm	Trend
307,1512	0,60	HMDB0241810	4-Amino-3-hydroxybutanoylcarnitine	47,1	0,53	▲
231,0617	0,60	HMDB0258248	serine glutamate	48,3	-2,28	▼
130,0517	0,69	HMDB0011614	7-Methyladenine	37,6	-3,86	▲
180,0870	0,72	HMDB0246693	D-Glucamine	37,9	-4,10	▲
406,2075	0,76	HMDB0241132	3-Hydroxydecanedioylcarnitine	49	-2,03	▲
452,2614	0,76	HMDB0241947	N-Stearoyl Methionine	43,9	1,90	▼
273,1215	0,78	HMDB0253016	Glycinamide, glycyl-L-prolyl-	47	4,74	▼
262,0875	1,26	HMDB0028737	Asparaginyl-Methionine	39,5	3,13	▲
401,2101	1,93	HMDB0003128	Cortolone	40,1	0,17	▲
473,2058	2,08	HMDB0007855	LysoPA(18:1(9Z)/0:0)	38,2	-4,12	▲
230,1154	2,21	HMDB0028744	Asparaginyl-Valine	47,5	3,39	▲
267,0992	2,68	HMDB0028848	Glycyl-Phenylalanine	39,1	2,51	▲
339,0151	2,99	HMDB0001570	Thymidine 3',5'-cyclic monophosphate	43,7	-1,22	▼
231,1356	3,07	HMDB0029064	Threonylisoleucine	46,1	2,53	▼
594,2880	3,21	HMDB0304803	Leu-Arg-Asn-Arg	45,1	-0,74	▲
371,1936	3,28	HMDB0029010	Prolyl-Alanine	43,3	-0,04	▼
458,2440	3,29	HMDB0114759	LysoPA(20:3(8Z,11Z,14Z)/0:0)	44,4	-1,91	▼
561,2492	3,29	HMDB0304812	Val-Phe-Val-Tyr	48,5	1,32	▲
782,4280	3,36	HMDB0280927	PS(PGE2/14:1(9Z))	42,8	3,78	▲
597,2631	3,40	HMDB0000279	Saccharopine	43,2	1,05	▼
400,2342	3,48	HMDB0241254	2-Hydroxydodeca-4,6-dienoylcarnitine	40,6	0,24	▲
871,4414	3,48	HMDB0268688	PG(6 keto-PGF1alpha/16:1(9Z))	50,9	3,98	▲
1000,5059	3,52	HMDB0006973	CDP-DG(16:0/20:4(5Z,8Z,11Z,14Z))	51,9	-1,10	▲
930,4536	3,54	HMDB0282802	PS(6 keto-PGF1alpha/20:5(5Z,8Z,11Z,14Z,17Z))	53,5	-0,61	▼
955,3744	3,60	HMDB0273193	PGP(18:3(9Z,12Z,15Z)/20:5(7Z,9Z,11E,13E,17Z)-3OH(5,6,15))	44,1	-4,09	▲
880,4182	3,62	HMDB0282835	PS(20:5(5Z,8Z,11Z,14Z,17Z)/20:5(5Z,8Z,11Z,14Z,16E)-OH(18))	54,8	1,09	▲
861,3378	3,79	HMDB0253117	Hexa-His	46,3	-1,12	▲
536,2451	3,88	HMDB0000896	Taurodeoxycholic acid	44,8	-0,63	▼
227,1042	3,89	HMDB0028756	Aspartyl-Isoleucine	45,2	1,76	▼

403,2185	3,91	HMDB0242068	N-Eicosapentaenoyl Cysteine	46,4	-2,98	▼
956,4398	3,96	HMDB0275027	PGP(i-14:0/LTE4)	51,8	3,42	▲
974,2871	4,13	HMDB0062426	5Z-tetradecenoyl-CoA	39,4	-3,65	▲
1006,4647	4,15	HMDB0247699	Pglu-Leu-Thr-Phe-Thr-Ser-Ser-Trp-GlyNH2	50,7	0,72	▲
1100,5223	4,15	HMDB0293861	CDP-DG(22:6(4Z,7Z,10Z,13E,15E,19Z)-OH(17)/i-17:0)	54,6	-0,73	▲
999,4627	4,18	HMDB0278242	PI(PGJ2/22:5(7Z,10Z,13Z,16Z,19Z))	49,5	-1,62	▲
447,0211	4,20	HMDB0001274	dTDP	39,4	-0,13	▼
500,2743	4,22	HMDB0011475	LysoPE(0:0/18:1(11Z))	49,7	-3,27	▲
444,1270	4,22	HMDB0060454	Chondroitin D-glucuronate	43,9	-2,07	▼
1078,4972	4,24	HMDB0293747	CDP-DG(20:4(6E,8Z,11Z,13E)-2OH(5S,15S)/i-16:0)	48,5	-4,93	▲
419,2411	4,32	HMDB0013010	N-Heptanoylglycine	42,5	3,12	▼
344,1697	4,34	HMDB0253872	L-Arginine, L-asparaginylglycyl-	47,7	2,60	▼
1128,4917	4,41	HMDB0280630	PIP(LTE4/22:5(4Z,7Z,10Z,13Z,16Z))	49,4	2,35	▲
920,4461	4,43	HMDB0116052	CDP-DG(18:2(9Z,11Z)/i-12:0)	44,1	1,84	▲
611,3130	4,43	HMDB0266643	PA(8:0/18:1(12Z)-O(9S,10R))	42,6	1,57	▲
667,3379	4,44	HMDB0267406	PA(18:3(9,11,15)-OH(13)/i-12:0)	52,8	-0,63	▼
825,4811	4,50	HMDB0261639	PE(6 keto-PGF1alpha/18:3(9Z,12Z,15Z))	53,1	0,43	▲
901,4755	4,53	HMDB0276265	PI(6 keto-PGF1alpha/16:2(9Z,12Z))	52,4	3,83	▲
932,4423	4,56	HMDB0292795	CDP-DG(a-13:0/18:1(12Z)-O(9S,10R))	48,5	-2,26	▲
1103,5447	4,59	HMDB0291730	CDP-DG(PGF2alpha/20:1(11Z))	51,2	-2,59	▼
616,3277	4,65	HMDB0288875	PC(20:4(6E,8Z,11Z,13E)-2OH(5S,15S)/2:0)	48,9	3,41	▲
759,3983	4,66	HMDB0267604	PA(22:6(4Z,7Z,11E,13Z,15E,19Z)-2OH(10S,17)/i-14:0)	51,2	-3,68	▲
963,4647	4,66	HMDB0293522	CDP-DG(i-14:0/18:1(12Z)-O(9S,10R))	51	0,90	▲
486,2592	4,71	HMDB0010380	LysoPC(14:1(9Z)/0:0)	45,7	-2,20	▼
472,2471	4,71	HMDB0011510	LysoPE(18:4(6Z,9Z,12Z,15Z)/0:0)	42,9	0,37	▲
300,1939	4,71	HMDB0028945	Lysylarginine	48,4	4,57	▲
812,3201	4,71	HMDB0251612	Dpp-II	45,1	4,54	▲
961,4613	4,73	HMDB0281872	PS(18:3(9Z,12Z,15Z)/LTE4)	50	-1,88	▲
1146,5315	4,73	HMDB0291938	CDP-DG(PGF2alpha/20:3(5Z,8Z,11Z))	54,4	2,69	▲
512,3051	4,75	HMDB0242023	N-Docosahexaenoyl Tryptophan	42,7	-0,61	▲
1018,5140	4,78	HMDB0291129	CDP-DG(18:1(11Z)/18:1(12Z)-2OH(9,10))	44,5	-3,47	▼
455,2495	4,80	HMDB0000907	Sulfolithocholic acid	42,4	4,75	▼
897,5407	4,80	HMDB0284783	PE(6 keto-PGF1alpha/DiMe(11,5))	53,2	2,71	▲

650,2940	4,84	HMDB0250699	Cys-Arg-Glu-Lys-Ala	50,5	0,50	▲
528,2975	4,85	HMDB0241822	(5Z)-7-[(1R,2R,3R)-3-Hydroxy-2-[(1E,3S)-3-hydroxy-5-phenylpent-1-en-1-yl]-5-oxocyclopentyl]hept-5-enoylcarnitine	50,7	1,62	▼
656,3906	4,89	HMDB0012342	PS(14:1(9Z)/14:1(9Z))	51,1	-4,02	▲
962,5242	4,89	HMDB0270149	PG(LTE4/22:4(7Z,10Z,13Z,16Z))	53,8	2,01	▼
1077,5078	4,91	HMDB0279757	PIP(PGF1alpha/20:2(11Z,14Z))	53,6	-1,03	▲
1187,6229	4,96	HMDB0259343	Tyr-ile-gly-ser-arg	42,9	4,28	▲
732,4396	4,96	HMDB0265226	PA(18:3(9,11,15)-OH(13)/20:4(5Z,8Z,11Z,14Z))	53,1	1,95	▼
970,4550	4,97	HMDB0274404	PGP(LTE4/a-15:0)	43,9	2,93	▲
582,3231	5,01	HMDB0288806	PC(2:0/20:4(5Z,8Z,11Z,14Z)-OH(20))	46,6	4,97	▼
783,4081	5,03	HMDB0264348	PA(18:4(6Z,9Z,12Z,15Z)/5-iso PGF2VI)	49,4	-1,16	▲
1141,5955	5,09	HMDB0013046	Psychosine sulfate	38,6	-2,25	▲
314,2094	5,09	HMDB0094676	Val-Val-Val	47,5	2,63	▼
467,1954	5,14	HMDB0114743	LysoPA(18:3(6Z,9Z,12Z)/0:0)	45,9	-3,89	▼
760,3942	5,16	HMDB0260741	PE(20:5(6E,8Z,11Z,14Z,17Z)-OH(5)/14:1(9Z))	50,6	-2,61	▲
362,2077	5,28	HMDB0304805	Leu-Pro-Ile	49,9	4,57	▲
931,4727	5,32	HMDB0273364	PGP(PGJ2/20:2(11Z,14Z))	55,7	-1,69	▲
517,2412	5,33	HMDB0245688	25-Hydroxyvitamin D3 3-sulfate ester	41,7	3,40	▼
1023,5842	5,42	HMDB0284476	PE(24:0/LTE4)	49,3	-3,86	▲
540,3013	5,44	HMDB0242415	Deoxycholylcysteine	39,5	2,57	▲
1140,5690	5,45	HMDB0293094	CDP-DG(20:3(8Z,11Z,14Z)-2OH(5,6)/a-21:0)	50,2	1,47	▲
876,4427	5,47	HMDB0281879	PS(18:3(9Z,12Z,15Z)/20:3(8Z,11Z,14Z)-2OH(5,6))	51,1	-0,87	▲
995,4531	5,49	HMDB0278631	PIP(16:2(9Z,12Z)/20:4(5Z,8Z,11Z,14Z)-OH(19S))	47,5	-0,96	▲
619,1943	5,51	HMDB0245624	21H-Biline-8,12-dipropanoicacid, 3,18-diethenyl-1,19,22,24-tetrahydro-2,7,13,17-tetramethyl-1,19-dioxo-	42,6	-3,60	▲
1120,5432	5,54	HMDB0293117	CDP-DG(a-21:0/20:5(6E,8Z,11Z,14Z,17Z)-OH(5))	48,7	1,82	▲
1130,5249	5,58	HMDB0291776	CDP-DG(22:6(5Z,7Z,10Z,13Z,16Z,19Z)-OH(4)/20:1(11Z))	54,7	-0,57	▲
503,2523	5,60	HMDB0062315	1-eicosanoyl-glycero-3-phosphate	37,3	-4,73	▼
576,3335	5,60	HMDB0288856	PC(2:0/18:1(12Z)-2OH(9,10))	42,5	4,78	▼
902,3561	5,64	HMDB0259306	TRYPSINOGEN	43,2	2,56	▼
840,4451	5,66	HMDB0281795	PS(20:5(5Z,8Z,11Z,14Z,16E)-OH(18R)/18:3(6Z,9Z,12Z))	55,4	2,19	▼
973,4666	5,67	HMDB0278505	PIP(16:0/18:3(9,11,15)-OH(13))	44,7	-3,26	▲
356,2559	5,79	HMDB0094648	Leu-Leu-Leu	40,7	1,26	▲
775,4706	5,79	HMDB0263580	PA(20:3(8Z,11Z,14Z)-O(5,6)/18:0)	48,4	2,67	▼

528,3306	5,83	HMDB0242380	Cholylvaline	41,8	-0,12	▼
558,3004	5,83	HMDB0242435	Deoxycholylmethionine	40,1	-4,08	▼
1047,5177	5,90	HMDB0280212	PIP(PGJ2/22:2(13Z,16Z))	52,3	-3,81	▲
945,5069	5,97	HMDB0276124	PI(16:0/PGF1alpha)	54	-4,76	▼
993,5068	5,99	HMDB0275642	PGP(i-20:0/6 keto-PGF1alpha)	40,8	-1,93	▼
845,4388	6,05	HMDB0265174	PA(TXB2/20:4(5Z,8Z,11Z,14Z))	52,1	1,28	▲
850,4328	6,24	HMDB0264338	PA(18:4(6Z,9Z,12Z,15Z)/LTE4)	48,1	-0,70	▲
1152,5355	6,31	HMDB0293121	CDP-DG(a-21:0/20:5(7Z,9Z,11E,13E,17Z)-3OH(5,6,15))	46,3	3,96	▲
796,3875	6,33	HMDB0262781	PA(LTE4/12:0)	47,7	4,36	▲
1018,4805	6,34	HMDB0292991	CDP-DG(a-17:0/5-iso PGF2VI)	45,8	-0,67	▲
604,3345	6,36	HMDB0012937	Dynorphin B (6-9)	44,5	3,36	▲
843,4605	6,45	HMDB0259047	Thrombin receptor peptide sfllrnp	49,5	-1,61	▲
512,2684	6,65	HMDB0260480	MG(LTE4/0:0/0:0)	42,7	-0,61	▼
866,5161	6,93	HMDB0281360	PS(18:0/20:3(8Z,11Z,14Z)-2OH(5,6))	41,1	-0,43	▲
498,2870	7,07	HMDB0000896	Taurodeoxycholic acid	39,1	-4,88	▼
514,2843	7,11	HMDB0000036	Taurocholic acid	43,9	-0,20	▼
498,2887	7,92	HMDB0000896	Taurodeoxycholic acid	40,4	-1,54	▼
566,3447	8,61	HMDB0002815	LysoPC(18:1(9Z)/0:0)	37,5	-3,17	▼
295,1382	9,20	HMDB0259738	Val-Cit	40,8	-2,19	▼
687,5440	9,36	HMDB0240617	SM(d16:1/17:0)	44,1	-0,88	▼
799,5277	9,39	HMDB0265733	PA(22:6(4Z,8Z,10Z,13Z,16Z,19Z)-OH(7)/22:1(13Z))	49,3	-0,74	▼
737,5359	9,39	HMDB0296506	DG(PGJ2/0:0/21:0)	49,6	2,95	▼
452,2787	9,50	HMDB0296941	DG(2:0/0:0/20:3(8Z,11Z,14Z)-2OH(5,6))	50,4	-0,55	▼
773,5781	9,53	HMDB0290687	SM(d19:1/18:1(12Z)-2OH(9,10))	45,7	-4,29	▼
540,3311	9,60	HMDB0243971	1-Nonadecanoyl-glycero-3-phosphoethanolamine	53,2	0,85	▼
806,5333	9,60	HMDB0284080	PE(22:4(7Z,10Z,13Z,16Z)/18:1(12Z)-2OH(9,10))	55,9	-1,03	▼
902,5206	9,73	HMDB0061606	PS(MonoMe(11,5)/DiMe(11,3))	46,5	4,70	▼
745,5508	10,14	HMDB0240638	SM(d18:2(4E,14Z)/16:0)	46,4	1,02	▲
685,5284	10,14	HMDB0240677	SM(d17:2(4E,8Z)/16:0)	41,5	-0,92	▲
209,1551	10,32	HMDB0035881	11-Hydroxy-9-tridecenoic acid	42,8	1,54	▼

Table S5: List of accepted identifications between control (HFHSD) and HFHSD MOE groups of HPI in ESI^- of plasma samples.

m/z	RT (min)	HMDB ID	Accepted Description	Score	ppm	Trend
205,0831	0,65	HMDB0257621	(2S,3R)-2-Amino-3-[(2S)-2-amino-3-hydroxypropanoyl]oxybutanoic acid	43,2	0,66	▼
347,1211	0,81	HMDB0255140	n-formimidoyl-glutamic acid	41,9	0,7	▼
217,0838	0,83	HMDB0006248	gamma-Glutamylalanine	44,6	3,82	▼
285,1034	0,88	HMDB0028891	Histidylmethionine	44,8	2,59	▲
555,1558	0,98	HMDB0255306	N7-(2-((Hydroxyethyl)thio)ethyl)guanine	40,8	-0,84	▲
308,1619	1,36	HMDB0028963	Lysyltyrosine	45	0,89	▼
930,4509	3,54	HMDB0282802	PS(6 keto-PGF1alpha/20:5(5Z,8Z,11Z,14Z,17Z))	53,7	-3,52	▼
755,3889	3,69	HMDB0001038	Angiotensin IV	54,3	0,31	▼
1090,5272	3,69	HMDB0116003	CDP-DG(20:4(8Z,11Z,14Z,17Z)/20:1(11Z))	50	-3,22	▼
322,1413	3,79	HMDB0241687	2-Hydroxyhept-5-enoylcarnitine	42	-4,74	▼
1002,5337	3,88	HMDB0269630	PG(LTE4/20:2(11Z,14Z))	46,8	-4,77	▼
227,1042	4	HMDB0011172	gamma-Glutamylvaline	46,7	2,08	▼
687,3639	4,02	HMDB0262800	PA(12:0/18:1(12Z)-2OH(9,10))	48,3	-0,98	▲
1047,448	4,02	HMDB0279274	PIP(6 keto-PGF1alpha/18:3(6Z,9Z,12Z))	49,8	1,48	▼
984,472	4,08	HMDB0116080	CDP-DG(a-15:0/18:2(9Z,11Z))	52,7	-1,33	▼
953,4994	4,08	HMDB0276745	PI(18:2(9Z,12Z)/PGD1)	52,8	-1,63	▼
311,1075	4,22	HMDB0028985	Methionyl-Tyrosine	43,9	1,24	▲
458,2612	4,24	HMDB0013253	N-Acetylhistamine	40,9	-4,76	▼
669,393	4,27	HMDB0114854	PA(16:1(9Z)/15:0)	47,2	4,18	▲
235,1094	4,32	HMDB0029443	L-Pyridosine	55,3	2,38	▲
993,5074	4,39	HMDB0275643	PGP(6 keto-PGF1alpha/i-20:0)	53,9	-1,31	▼
908,4339	4,46	HMDB0281773	PS(TXB2/18:3(6Z,9Z,12Z))	56,9	0,65	▼
960,4485	4,48	HMDB0116265	CDP-DG(i-18:0/i-12:0)	47,7	-4,18	▲

290,115	4,63	HMDB0253030	Tyrosyl-alanyl-glycine	47,3	1,29	▲
933,5069	4,75	HMDB0270142	PG(22:4(7Z,10Z,13Z,16Z)/6 keto-PGF1alpha)	53,5	-4,57	▲
479,2161	4,77	HMDB0114742	LysoPA(20:4(8Z,11Z,14Z,17Z)/0:0)	48,6	-4,06	▼
512,3077	4,77	HMDB0242023	N-Docosahexaenoyl Tryptophan	47,3	4,32	▲
415,2316	4,78	HMDB0252758	Glutamylleucylarginine	45,7	1,21	▼
599,3385	4,78	HMDB0258027	(8S,9R,10R,13S,14S)-10,13-Dimethyl-4,5,6,7,8,9,12,14,15,16-decahydrocyclopenta[a]phenanthrene-3,11,17-trione	46,3	1,12	▼
671,4077	4,84	HMDB0114984	PA(18:3(6Z,9Z,12Z)/18:4(6Z,9Z,12Z,15Z))	51,7	-0,73	▼
833,4847	4,85	HMDB0268698	PG(TXB2/16:1(9Z))	50,5	3,05	▼
528,3017	4,87	HMDB0000722	Lithocholyltaurine	40,7	3,5	▼
754,4442	4,87	HMDB0261763	PE(20:4(5Z,8Z,11Z,13E)+=O(15)/18:4(6Z,9Z,12Z,15Z))	54,7	-1,48	▲
962,4899	4,91	HMDB0010030	PIP(22:5(7Z,10Z,13Z,16Z,19Z)/16:0)	37,6	-3,95	▼
947,5274	4,92	HMDB0276423	PI(22:6(4Z,7Z,10Z,13E,15E,19Z)-OH(17)/18:0)	50,6	0,78	▼
891,4689	4,92	HMDB0276788	PI(PGJ2/18:3(6Z,9Z,12Z))	55	2,6	▼
1022,4788	4,92	HMDB0293561	CDP-DG(PGD1/i-14:0)	51,8	2,6	▼
364,1905	4,97	HMDB0241139	4-Oxodecanoylcarnitine	46,1	2,52	▼
392,1838	4,97	HMDB0241249	3-Hydroxydodeca-7,10-dienoylcarnitine	45,6	-1,93	▼
824,3927	4,97	HMDB0262884	PA(13:0/LTE4)	47,9	-2,21	▼
846,4793	4,99	HMDB0281253	PS(TXB2/16:1(9Z))	51,7	2,28	▼
516,2978	5,01	HMDB0241812	(4Z,7Z,10S,11E)-10-Hydroperoxy-12-[(1R,4S,5S,6R)-6-[(2Z)-pent-2-en-1-yl]-2,3-dioxabicyclo[2.2.1]heptan-5-yl]dodeca-4,7,11-trienoylcarnitine	46,4	2,16	▼
1193,7175	5,01	HMDB0243883	1-Heneicosanoyl-glycero-3-phosphoserine	46,9	-3,16	▼
761,4424	5,03	HMDB0264583	PA(20:5(7Z,9Z,11E,13E,17Z)-3OH(5,6,15)/19:2(10Z,13Z))	52,3	3,23	▼
314,2092	5,11	HMDB0094676	Val-Val-Val	48,6	2,25	▼
629,4205	5,11	HMDB0294699	DG(12:0/0:0/20:3(8Z,11Z,14Z)-2OH(5,6))	46,2	2,66	▼

837,3725	5,12	HMDB0274324	PGP(a-13:0/18:1(9Z)-O(12,13))	49,3	-0,29	▲
569,3036	5,14	HMDB0003577	VPGPR Enterostatin	48,9	-3,17	▼
820,4566	5,18	HMDB0261447	PE(5-iso PGF2VI/18:2(9Z,12Z))	54,5	3,74	▼
904,4341	5,19	HMDB0281970	PS(6 keto-PGF1alpha/18:4(6Z,9Z,12Z,15Z))	45,4	-4,94	▲
775,4141	5,23	HMDB0271131	PG(i-12:0/22:6(4Z,7Z,10Z,13E,15E,19Z)-OH(17))	51,8	-3,59	▼
957,4525	5,28	HMDB0276942	PI(22:6(4Z,7Z,10Z,13E,15E,19Z)-OH(17)/18:3(9Z,12Z,15Z))	52,2	-1,27	▼
369,2872	5,4	HMDB0013329	trans-2-Tetradecenoylcarnitine	41,7	-3,43	▼
627,3232	5,45	HMDB0248672	Astacin	42,6	-2,52	▼
595,3804	5,58	HMDB0244285	(6S,3'S,5'R,6'R)-6,3',5'-Trihydroxy-4,5,6',7'-tetradehydro-7,8,5',6'-tetrahydro-beta,beta-carotene-3,8-dione	53,6	1,81	▼
628,2013	5,6	HMDB0241922	N-Palmitoyl Aspartic acid	45,4	-1,51	▲
840,444	5,66	HMDB0281795	PS(20:5(5Z,8Z,11Z,14Z,16E)-OH(18R)/18:3(6Z,9Z,12Z))	55	0,87	▼
775,4704	5,79	HMDB0115373	PA(22:5(4Z,7Z,10Z,13Z,16Z)/22:6(4Z,7Z,10Z,13Z,16Z,19Z))	48,9	-0,48	▼
627,3894	5,86	HMDB0294754	DG(20:5(7Z,9Z,11E,13E,17Z)-3OH(5,6,15)/12:0/0:0)	43,2	2,51	▼
945,5051	5,97	HMDB0262269	PE(LTE4/20:3(8Z,11Z,14Z))	53,8	0,64	▼
845,4394	6,05	HMDB0272401	PGP(20:4(5Z,8Z,11Z,14Z)-OH(20)/16:1(9Z))	50,1	2,11	▲
1090,0031	6,17	HMDB0069208	TG(22:0/21:0/i-22:0)	35,4	2,37	▼
986,5304	6,17	HMDB0116058	CDP-DG(18:2(9Z,11Z)/i-18:0)	48,8	2,61	▼
968,4954	6,31	HMDB0012024	Ganglioside GT2 (d18:0/16:0)	38,7	0,83	▼
604,3305	6,36	HMDB0012937	Dynorphin B (6-9)	43,2	-3,29	▼
641,4205	6,38	HMDB0263011	PA(18:1(12Z)-O(9S,10R)/14:0)	40,4	2,58	▼
962,5306	6,46	HMDB0116279	CDP-DG(i-19:0/a-15:0)	51,2	2,91	▼
514,2784	6,55	HMDB0241876	(5Z)-7-[(1R,2R,5S)-5-Hydroxy-2-[(1E,3S,5Z)-3-hydroxyocta-1,5-dien-1-yl]-3-oxocyclopentyl]hept-5-enoylcarnitine	47,1	-0,37	▼
1060,5331	6,61	HMDB0291002	CDP-DG(PGF2alpha/18:0)	52,4	4,61	▼
514,2842	7,13	HMDB0257923	2-[(3alpha,7alpha,12alpha-Trihydroxy-24-oxocholane-24-yl)amino]ethanesulfonic acid	42,9	-0,46	▼

309,1682	8,71	HMDB0252828	Glycylprolylarginine	44,2	0,38	▲
763,3995	8,74	HMDB0263233	PA(20:5(7Z,9Z,11E,13E,17Z)-3OH(5,6,15)/15:0)	42,8	4,94	▲
524,2785	9,18	HMDB0241623	(4Z,8Z,10Z,13Z,16Z,19Z)-7-Hydroxydocosa-4,8,10,13,16,19-hexaenoylcarnitine	44,8	0,23	▲
795,5623	9,22	HMDB0290620	SM(d19:0/PGE2)	52,4	-4,26	▼
974,5269	9,36	HMDB0274646	PGP(22:6(5Z,8E,10Z,13Z,15E,19Z)-2OH(7S, 17S)/a-21:0)	41,6	-3,25	▼
747,5649	9,38	HMDB0013464	SM(d18:0/16:1(9Z))	46,9	-1,25	▼
687,5441	9,38	HMDB0240608	SM(d18:1/15:0)	38,1	-0,82	▼
918,5532	9,68	HMDB0283153	PS(22:6(5Z,8E,10Z,13Z,15E,19Z)-2OH(7S, 17S)/22:2(13Z,16Z))	47,3	3,33	▲
607,4169	10,06	HMDB0304785	Lysyl-Lysine	40,9	3,83	▼
494,3255	10,11	HMDB0241560	9-HydroxyNonadecanoylcarnitine	47,1	0,42	▲
745,55	10,14	HMDB0240638	SM(d18:2(4E,14Z)/16:0)	50,9	-0,2	▲
599,3173	10,4	HMDB0240261	LysoPI(18:0/0:0)	41,4	-4,87	▼
568,3621	10,49	HMDB0010384	LysoPC(18:0/0:0)	42,9	0,2	▲
508,3408	10,49	HMDB0012108	LysoPC(17:0/0:0)	54,2	-0,19	▲

Table S6: List of accepted identifications between control (SD) and HFHSD groups of HPO in ESI$^-$ of brain samples.

m/z	RT (min)	HMDB ID	Accepted Description	Score	ppm	Trend
305,067	4,39	HMDB0028755	Aspartyl-Histidine	43,6	4,18	▲
288,088	4,42	HMDB0253186	Histidylprolineamide	45,1	4,68	▲
347,1349	4,47	HMDB0028997	Phenylalanylhistidine	45,2	-3,93	▼
1058,5172	4,65	HMDB0280316	PIP(PGJ2/22:3(10Z,13Z,16Z))	41,8	2,22	▼
291,0517	4,69	HMDB0006809	Nicotinic acid ribonucleoside	42,8	0,57	▼
360,2381	4,72	HMDB0242059	N-Myristoyl Serine	45,9	-3,27	▼
261,0884	4,91	HMDB0028991	Phenylalanylaspartic acid	41,7	1,22	▼
237,1126	9,31	HMDB0003334	Symmetric dimethylarginine	46,1	1,19	▼
367,2002	12,41	HMDB0241263	Dodeca-4,9-dienedioylcarnitine	36,8	-2,17	▲
513,3816	12,78	HMDB0297171	DG(8:0/18:1(12Z)-2OH(9,10)/0:0)	38,7	3,75	▲
636,4948	13,09	HMDB0249815	Ceramide AP	40,7	-4,46	▲
452,2782	13,26	HMDB0011473	LysoPE(0:0/16:0)	45,1	-0,24	▲
444,275	13,41	HMDB0242007	N-Docosahexaenoyl Alanine	40	-1,36	▲
544,2668	13,45	HMDB0061694	1-Oleoylglycerophosphoserine	53,3	2,1	▲
489,2794	13,46	HMDB0012556	13'-Carboxy-alpha-tocotrienol	41,4	3,83	▲
484,3628	13,63	HMDB0241564	(10Z)-Nonadec-10-enoylcarnitine	40,2	-3,61	▲
436,2821	13,67	HMDB0241434	5-Hydroxypentadecanoylcarnitine	44,9	-3,59	▲
422,2506	13,89	HMDB0241930	N-Palmitoyl Methionine	56,5	1,18	▲
462,2989	13,96	HMDB0240599	LysoPE(P-18:1(9Z)/0:0)	56,8	-0,24	▲
610,54	14,50	HMDB0011763	Cer(d18:0/18:1(9Z))	48,4	-2,82	▲
638,5703	14,50	HMDB0240682	Cer(d16:1/22:0)	46,3	-4,45	▲
250,1458	14,60	HMDB0241690	Hepta-2,4-dienoylcarnitine	56,2	3,28	▼
480,3091	14,63	HMDB0011129	LysoPE(0:0/18:0)	53,5	-0,97	▲
564,5357	14,87	HMDB0011762	Cer(d18:0/18:1(11Z))	38,7	-0,74	▲
464,3141	15,06	HMDB0241515	12-Hydroxyheptadecanoylcarnitine	41,6	-1,71	▲

Table S7: List of accepted identifications between control (HFHSD) and HFHSD MOE groups of HPO in ESI$^-$ of brain samples.

m/z	RT (min)	HMDB ID	Accepted Description	Score	ppm	Trend
154,0624	0,55	HMDB0004225	2-Oxoarginine	45,2	1,24	▲
286,1879	0,65	HMDB0028712	Arginylisoleucine	41,3	-1,92	▼
133,0144	0,70	HMDB0000744	Malic acid	39,4	1,19	▲
229,1557	3,33	HMDB0029130	Valylisoleucine	48,5	-0,25	▼
243,1717	3,86	HMDB0253148	(2S)-6-Amino-2-(hexanoylamino) hexanoic Acid	44,1	1,03	▼
277,1559	4,20	HMDB0013243	Leucylphenylalanine	44,3	0,37	▼
291,0517	4,69	HMDB0006809	Nicotinic acid ribonucleoside	42,8	0,57	▲
261,0884	4,91	HMDB0028991	Phenylalanylaspartic acid	41,7	1,22	▲

445,1869	7,71	HMDB0004483	Estrone glucuronide	41,7	0,23	▲
804,5751	12,05	HMDB0286158	PC(18:1(12Z)-2OH(9,10)/17:0)	51,6	-1,12	▲
515,3619	12,10	HMDB0242086	N-Nervonoyl Asparagine	51,9	-0,53	▼
524,2784	12,68	HMDB0241622	(5Z,7Z,10Z,13Z,16Z,19Z)-4-Hydroxydocosa-5,7,10,13,16,19-hexaenoylcarnitine	47,8	-0,06	▲
850,557	12,78	HMDB0284014	PE(22:2(13Z,16Z)/PGE1)	51,8	-3,81	▼
513,3816	12,78	HMDB0297171	DG(8:0/18:1(12Z)-2OH(9,10)/0:0)	38,7	3,75	▼
636,4948	13,09	HMDB0249815	Ceramide AP	40,7	-4,46	▼
312,2544	13,10	HMDB0013034	Palmitoylglycine	48	-0,15	▼
452,2782	13,26	HMDB0011473	LysoPE(0:0/16:0)	45,1	-0,24	▲
571,2882	13,33	HMDB0000739	Isodesmosine	45,9	4,45	▲
484,3628	13,63	HMDB0241564	(10Z)-Nonadec-10-enoylcarnitine	40,2	-3,61	▼
645,3271	13,84	HMDB0240261	LysoPI(18:0/0:0)	38,7	2,32	▼
422,2506	13,89	HMDB0241930	N-Palmitoyl Methionine	56,5	1,18	▼
391,156	13,91	HMDB0186954	4-Androsten-3beta,17beta-diol 3-sulfate	52,9	-0,27	▼
800,6264	14,50	HMDB0010710	Galactosylceramide (d18:1/20:0)	44,4	0,95	▼
250,1458	14,60	HMDB0241690	Hepta-2,4-dienoylcarnitine	56,2	3,28	▲

Table S8: List of accepted identifications between control (HFHSD) and HFHSD MOE groups of HPI in ESI$^-$ of brain samples.

m/z	RT (min)	HMDB ID	Accepted Description	Score	ppm	Trend
920,6544	0,56	HMDB0288651	PC(18:1(12Z)-O(9S,10R)/24:0)	35,3	3,14	▲
289,0332	0,58	HMDB0001068	D-Sedoheptulose 7-phosphate	49,1	0,71	▲
199,001	0,60	HMDB0001321	D-Erythrose 4-phosphate	45,7	-1,4	▲
259,0227	0,60	HMDB0003971	Beta-D-Fructose 6-phosphate	48,6	1,18	▲
489,041	0,61	HMDB0000797	SAICAR	50,3	-4,61	▲
184,9855	0,61	HMDB0000807	3-Phosphoglyceric acid	42,9	-0,91	▲
292,922	0,61	HMDB0001429	Phosphate	39,8	-4,9	▲
239,0654	0,68	HMDB0029040	Serylhydroxyproline	39,9	2,22	▲
243,0624	0,68	HMDB0000767	Pseudouridine	46,3	0,45	▲
588,0743	0,73	HMDB0001095	GDP-L-fucose	39,3	-1,1	▲
362,0508	0,80	HMDB0240587	Guanosine 3'-monophosphate	46,5	0,24	▲
302,0518	0,84	HMDB0013220	Beta-Citryl-L-glutamic acid	43,4	0,03	▲
191,0196	0,84	HMDB0041862	D-Glucaro-1,4-lactone	44,4	-0,6	▲
242,1394	3,60	HMDB0241654	3-Hydroxyvalerylcarnitine	44,6	-1,29	▼
288,1451	3,65	HMDB0241656	4-Tiglylcarnitine	43,1	-0,51	▼
328,2479	4,20	HMDB0242060	N-Myristoyl Threonine	36,4	-4,44	▼
374,2541	4,20	HMDB0242060	N-Myristoyl Threonine	39,8	-2,09	▼
372,2381	4,44	HMDB0255637	Nitro-oleic acid	36,4	-3,34	▼
401,2588	4,72	HMDB0242080	N-Eicosapentaenoyl Threonine	37,3	1,5	▼
243,1602	5,28	HMDB0032248	11-Dodecenoic acid	48	-0,03	▼
358,259	5,32	HMDB0013164	2-Hydroxylauroylcarnitine	42,5	-2,48	▼
348,2306	6,27	HMDB0242034	N-Lauroyl Isoleucine	40,5	-1,55	▼
313,237	6,73	HMDB0030935	9,10,13-Trihydroxystearic acid	38,7	-4,17	▼
315,2534	6,93	HMDB0002259	Heptadecanoic acid	39,6	-2,39	▼
315,2538	7,29	HMDB0059633	(9S,10S)-9,10-dihydroxyoctadecanoate	43	-1,04	▼
309,1662	8,48	HMDB0252764	Glucitol-lysine	39,9	-1,65	▼
669,503	11,34	HMDB0256098	2,3-Dipalmitoyl-S-glycerylcysteine	43,3	1,84	▼
208,0728	12,34	HMDB0000014	Deoxycytidine	46,7	-0,06	▼

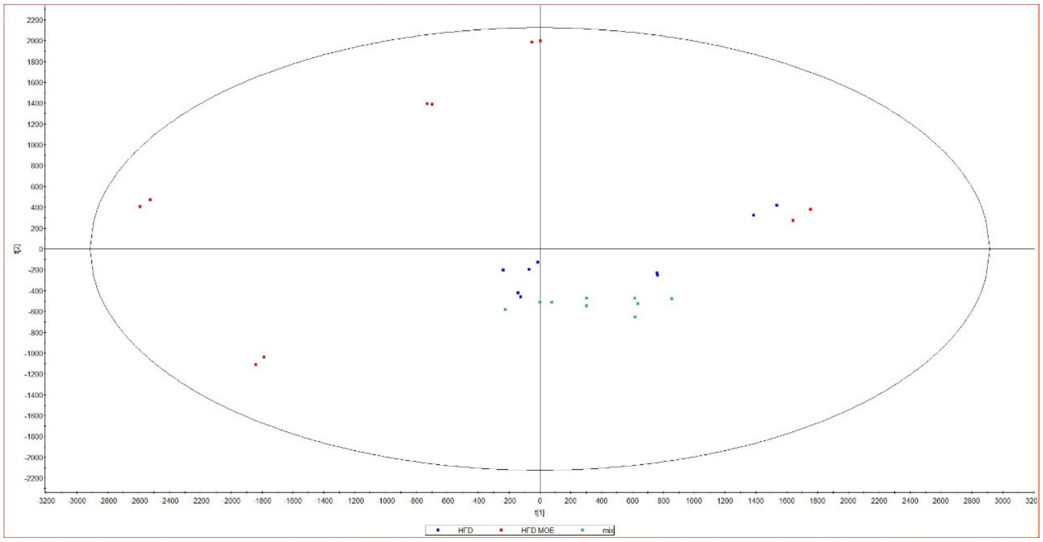

Figure S2: PCA containing HFHSD MOE compared to HFHSD of HPO in ESI$^-$

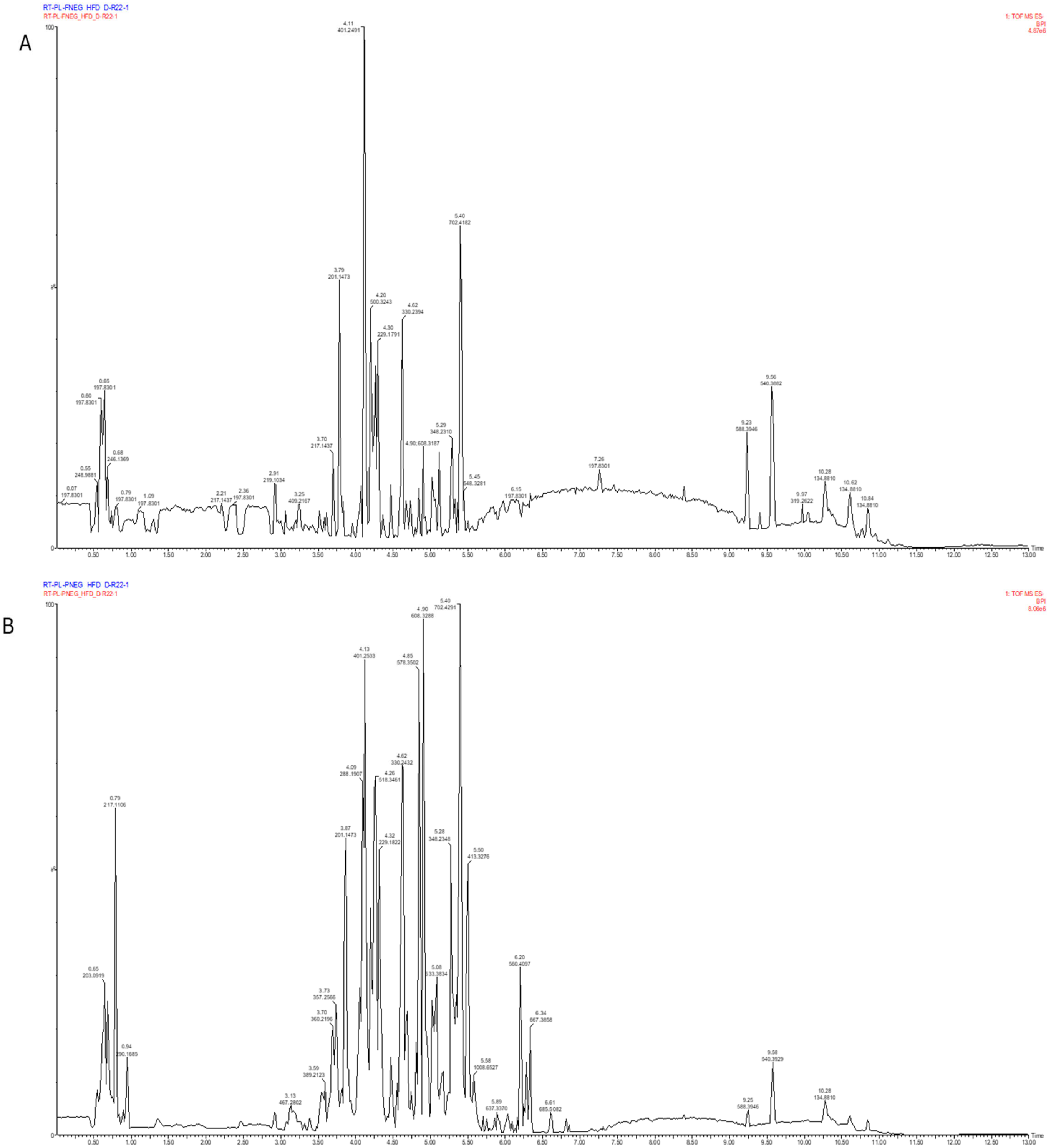

Figure S3: Representative pic base intensity chromatograms of (A) hydrophobic and (B) hydrophilic plasma samples

A

B

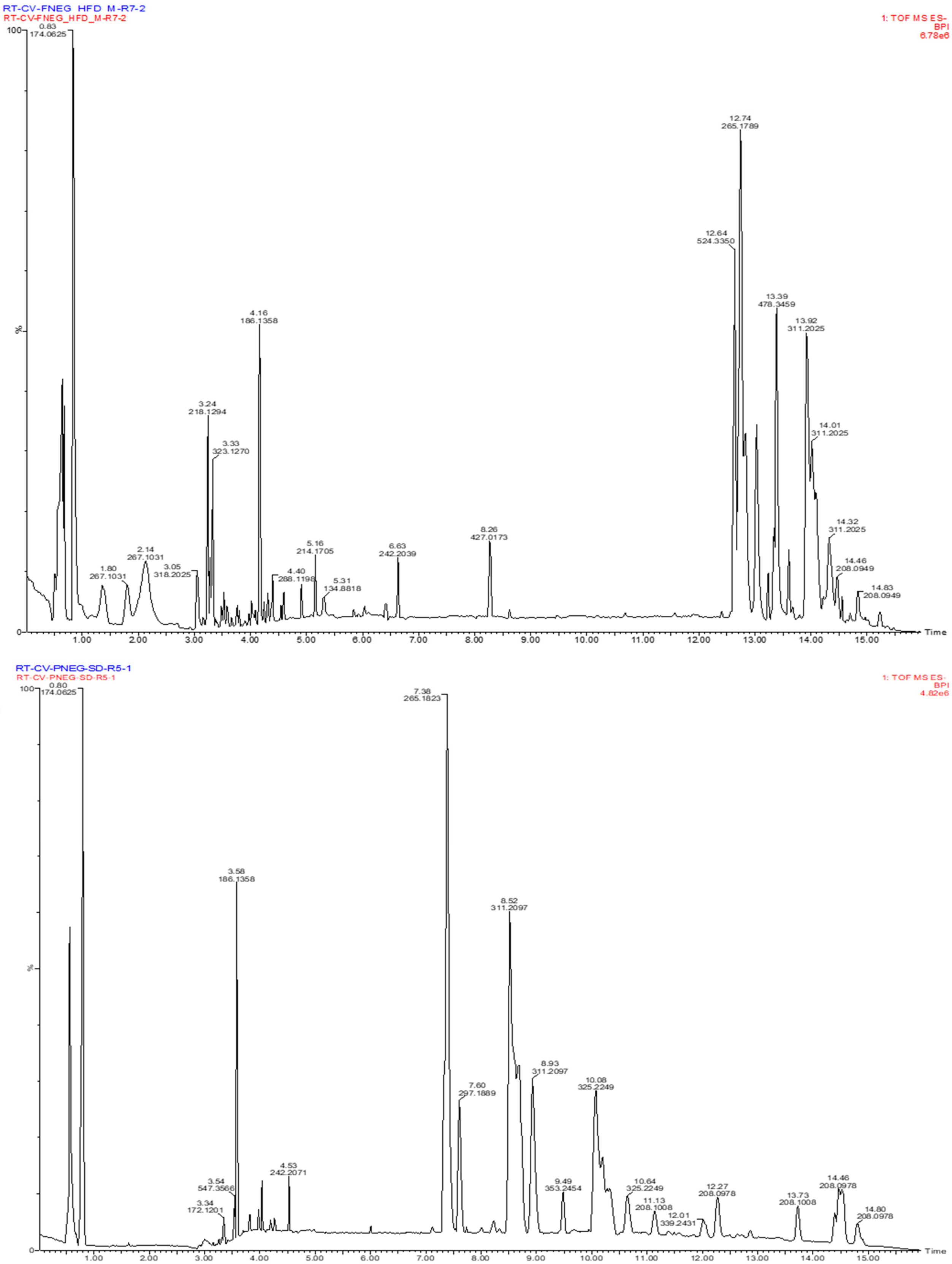

Figure S4: Representative pic base intensity chromatograms of (A) hydrophobic and (B) hydrophilic brain samples
